# Schistosoma japonicum histone acetyltransferase 1 (SjHAT1): A novel anti-schistosomal drug target

**DOI:** 10.1371/journal.ppat.1014334

**Published:** 2026-06-24

**Authors:** Jing Xu, Yu-Xin Wang, Ping Huang, Ya-Nan Zhang, Huan Sun, Ting-Zheng Zhan, Chao-Ming Xia

**Affiliations:** 1 Department of Parasitology, School of Basic Medical Sciences, Suzhou Medical College, Soochow University, Suzhou, Jiangsu, P. R. China; 2 MOE Key Laboratory of Geriatric Diseases and Immunology, Suzhou Key Laboratory of Pathogen Bioscience and Anti-infective Medicine, Suzhou Medical College, Soochow University, Suzhou, Jiangsu, P. R. China; 3 Key Laboratory of Basic Research on Regional Diseases (Guangxi Medical University), Education Department of Guangxi Zhuang Autonomous Region, Nanning, Guangxi, P. R. China; 4 Experimental Center of Suzhou Medical College, Soochow University, Suzhou, Jiangsu, P. R. China; 5 The Second Affiliated Hospital of Soochow University, Suzhou, Jiangsu, P. R. China; University of Wisconsin-Madison, UNITED STATES OF AMERICA

## Abstract

Schistosomiasis remains a critical global health issue, necessitating novel therapies due to emerging praziquantel resistance. We previously developed a patented praziquantel derivative, DW-3–15, which demonstrated potent broad-spectrum schistosomicidal activity through *Sj*HAT1 inhibition. Here, the full-length *Sj*HAT1 cDNA is cloned by rapid amplification of cDNA ends methods. The protein encoded by the cDNA retains conserved catalytic residues of acetyltransferases although sharing 34% identity with mammalian orthologs. Enzyme activity analysis reveals that recombinant *Sj*HAT1 exhibits 130 μU/mL histone acetyltransferase activity at 25°C for 60 min, and is completely inhibited by DW-3–15 at a concentration of 50 μM. However, DW-3–15 has no effect on the enzymatic activity of the human ortholog HAT1. Phylogenetic analyses place *Sj*HAT1 in a distinct clade, and molecular docking results indicate significant divergence compared to its human ortholog *Hs*HAT1. The expression profiling of *SjHAT1* reveals stage- and sex-specific patterns. Fluorescence *in situ* hybridization localizes *SjHAT1* predominantly in female vitellaria and male parenchyma near the gynecophoral canal. Knockdown of *SjHAT1* reduces worm survival by 57.5% *in vivo*, suppresses female oviposition by 90.8% *in vitro* and 79.2% *in vivo*, and disrupts ovarian and vitelline architecture. RNA-sequencing analysis reveals that *SjHAT1* knockdown disrupts β-alanyl-tryptamine (BATT) pheromone signaling via downregulation of *aromatic L-amino acid decarboxylase* (*AADC*) in males and *multidrug resistance-associated protein 4* (*MRP4*) in females. Given that BATT is essential for inducing female sexual development and egg production, the observed transcriptional dysregulation provides mechanistic evidence for disrupted intersexual communication. Our findings identify that *Sj*HAT1 is a central regulator of schistosome reproductive biology. These sex-specific mechanisms highlight *Sj*HAT1 as a potential therapeutic target for treatment and control of schistosomiasis.

## Author summary

Schistosomiasis is a neglected tropical disease caused by *Schistosoma* species. Reliance on the single drug praziquantel (PZQ) for treatment and control of schistosomiasis is not sustainable. Emerging drug resistance highlights the critical need for alternative treatments. In our previous studies, we developed a novel derivative of PZQ, designated DW-3–15, which exhibited broad-spectrum schistosomicidal activity against all developmental stages of *S. japonicum in vivo*, with notably potent efficacy against juveniles. Further, we observed synergistic effects when combining PZQ with DW-3–15 *in vitro* and *in vivo*, suggesting that there were distinct molecular targets for the two compounds. Later, we found that the therapeutic effect of DW-3–15 was mediated through downregulation of *Schistosoma japonicum* histone acetyltransferase 1 (*Sj*HAT1). Understanding the biological function played by *Sj*HAT1 is of great importance for developing novel anti-schistosomal drug targets. In this study, we find that *SjHAT1* is predominantly expressed in the vitellaria of female worms and in the parenchyma near the gynecophoral canal of male worms. The localization of *Sj*HAT1 in schistosomes indicates its significant role in the reproductive development and egg-laying processes of female worms. Eggs are the key pathogenic factors and are responsible for the transmission of schistosomiasis. Knockdown of *SjHAT1* significantly compromises worm survival and suppresses female oviposition. Transcriptomic analysis further reveals that *SjHAT1* silencing disrupts schistosome β-alanyl-tryptamine (BATT) pheromone signaling via two synergistic pathways. Specifically, male-specific suppression of *aromatic L-amino acid decarboxylase* (*AADC*) impairs BATT pheromone synthesis, thereby hindering female sexual maturity. Concurrently, female-specific downregulation of *multidrug resistance-associated protein 4* (*MRP4*) inhibits BATT signal transduction, synergistically blocking oviposition. As BATT is essential for inducing female sexual development and egg production, these findings establish *Sj*HAT1 as a central regulator of schistosome reproductive biology. We have validated *Sj*HAT1 as a promising druggable target through *in vitro* and *in vivo* investigations. Further studies are required to establish *Sj*HAT1 as a specific epigenetic regulator governing schistosome reproduction. The dual-sex regulatory mechanism provides a framework for understanding the fascinating sexual biology of schistosomes, which could lead to novel strategies for treatment and control of schistosomiasis.

## Introduction

Schistosomiasis is a neglected parasitic disease caused by infection with trematodes of the genus *Schistosoma*, mainly *Schistosoma mansoni*, *Schistosoma haematobium*, *Schistosoma japonicum*, *Schistosoma intercalatum*, *Schistosoma guineensis*, and *Schistosoma mekongi*. Current global data indicate that approximately 1 billion people are at risk of schistosomiasis, with more than 250 million people infected across 78 countries [1,2]. Annually, about 250,000 deaths are attributed to this disease [3]. In the absence of an effective vaccine, praziquantel (PZQ) is the only available drug to control this debilitating disease [4]. However, PZQ is ineffective against immature worms and does not prevent reinfection, and repeated use of drug monotherapy in many endemic regions has raised serious concerns about the emergence of drug resistance [5,6]. Therefore, developing alternative treatments and identifying novel drug targets for schistosomiasis remains crucial.

Currently, three strategies dominate the search for new anti-schistosomal drugs: (1) synthesis and evaluation of PZQ analogues, (2) design of new pharmacophores and (3) high-throughput screening of new active compounds [7]. While designing novel pharmacophores is challenging due to the still unclear mechanism of PZQ [8], the development of PZQ derivatives remains a primary approach for discovering alternative therapeutics. In prior work, our group hybridized the endoperoxide bridge pharmacophore of artesunate (which targets juvenile schistosomes) to PZQ (which targets adults) by inserting a hydroxyl group at the position C10 of the pharmacophore aromatic ring in PZQ, yielding a novel praziquantel derivative DW-3–15 (Patent No. ZL201110142538.2) [9]. DW-3–15 exhibited broad-spectrum schistosomicidal activity against all developmental stages of *S. japonicum in vivo*, with especially high efficacy against juveniles [10]. The anti-juvenile and anti-adult effects of DW-3–15 were consistently observed in commercially synthesized batches, confirming its structural stability. Moreover, DW-3–15 significantly reduced egg deposition by female worms and mitigated hepatic pathology, further supporting its therapeutic potential [11]. Notably, combining PZQ with DW-3–15 produced synergistic effects *in vitro* and *in vivo*; even at sub-therapeutic doses, the combination achieved efficacy comparable to or exceeding either monotherapy, suggesting the two compounds have distinct molecular targets [12].

To investigate the molecular mechanism of DW-3–15, we performed tandem mass tag (TMT) quantitative proteomics on treated worms. This analysis revealed that *S. japonicum* histone acetyltransferase 1 (*Sj*HAT1) was one of the most significantly downregulated proteins in female worms following *in viv*o DW-3–15 treatment [13]. HAT1 is a member of the GCN5-related N-acetyltransferases (GNAT) superfamily and is one of the first histone acetyltransferases discovered, primarily acetylating lysine 5 and lysine 12 of histone H4 (H4K5 and H4K12) [14]. HATs catalyze the transfer of acetyl groups to lysine residues on histones, thereby increasing chromatin accessibility to promote gene transcription [15]. This acetylation-mediated post-translational modiﬁcation plays crucial roles in the growth, development, and reproduction of schistosomes [16–19]. However, only few investigations on HAT inhibitors against schistosomes are currently available. Carneiro et al [18] demonstrated that histone acetylation by *Sm*CBP1 and *Sm*GCN5 in *S. mansoni* establishes an epigenetic state necessary for *Smp14* gene activation and eggshell formation; the HAT inhibitor PU139 prevents chromatin decondensation at the *Smp14* promoter, thereby impairing egg production and disrupting female reproductive development [18]. Furthermore, testing of the HAT inhibitors A485, C646, and curcumin identified curcumin as a potent schistosomicidal agent that dose-dependently suppresses *Sj*HAT1 expression. Notably, we also found that DW-3–15 could inhibit *Sj*HAT1 expression, implicating *Sj*HAT1 as a promising target mediating its anti-schistosomal effects [13].

Despite these findings, the biological roles of *Sj*HAT1 in *S. japonicum*, particularly its involvement in drug-mediated killing of worms and in regulating female reproductive activity, remain poorly characterized. In this study, we employed rapid amplification of cDNA ends (RACE) to systematically analyze the bioinformatics features of *Sj*HAT1. Subsequently, we investigated its functions both *in vitro* and *in vivo*. Finally, we utilized RNA sequencing (RNA-seq) to explore the regulatory role of *Sj*HAT1 in downstream signaling pathways, thereby establishing experimental and theoretical foundations for the development of *Sj*HAT1-targeted anti-schistosomal interventions.

## Results

### *Sj*HAT1 is a promising drug target against schistosomiasis

**Fig 1:**
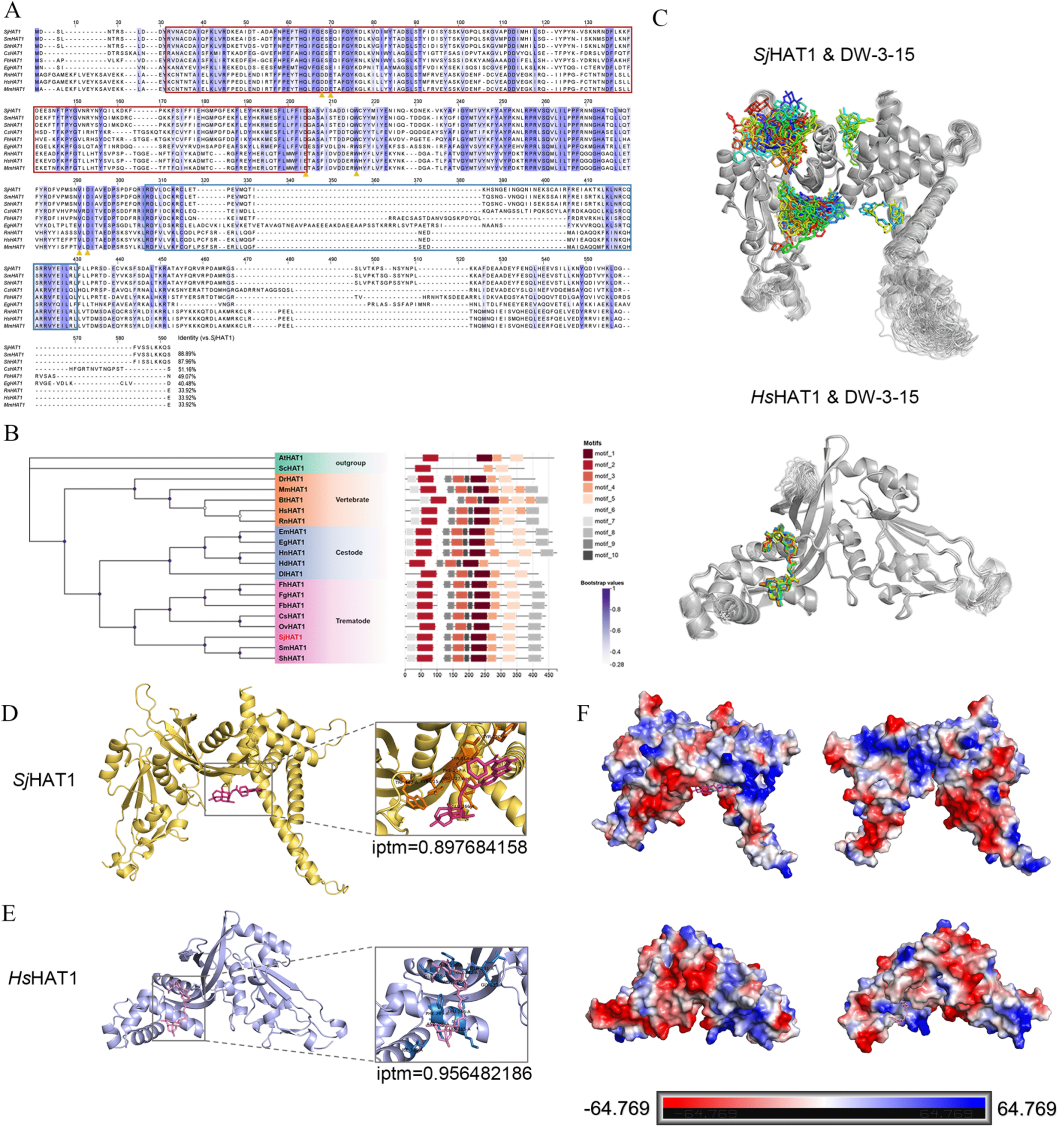
Multiple sequences alignment, phylogenetic analysis and molecular docking analysis of *Sj*HAT1. **(A)** Multiple sequences alignment of *Sj*HAT1. Red box indicates the N-terminal domain of HAT1, blue box indicates the C-terminal domain of HAT1, and yellow triangles highlight key amino acids experimentally validated to be associated with HAT1 protein activity in other species **(B)** phylogenetic analysis of *Sj*HAT1. *Sj*HAT1 is shown in red. The right panel illustrates the structural organization and phylogenetic distribution of conserved motifs within HAT1 orthologs across different species. **(C)** Computational characterization of *Sj*HAT1/*Hs*HAT1and DW-3-15 interactions using molecular docking and ensemble analysis. Structural ensemble analysis was performed on the top 100 docking poses ranked by interface-predicted TM (ipTM) score through a computational modeling framework. Ligand conformations were chromatically coded according to binding energy gradients, while target proteins *Sj*HAT1/*Hs*HAT1 were displayed as monochromatic surface representations with transparency-adjusted secondary structures. **(D)** Three-dimensional structural model of *Sj*HAT1 with the highest ipTM score and its ligand-binding site. The DW-3-15 ligand represented as magenta sticks. Close-up view of the ligand-binding pocket, highlighting critical interactions with key amino acid residues (Trp-187, Tyr-215, Phe-217, Tyr-218, Tyr-220, Arg-227 and Glu-266). **(E)** Three-dimensional structural model of *Hs*HAT1 with the highest ipTM score and its ligand-binding site. The DW-3-15 ligand represented as pink sticks. Close-up view of the ligand-binding pocket, highlighting critical interactions with key amino acid residues (Gly-230, Gln-231, Ala-235, Glu-239, Lys-265, Leu-266, Asp-268, Phe-269, Ile-315). **(F)** Comparison of surface electrostatic potential distributions between *Sj*HAT1 and *Hs*HAT1 Proteins. Red areas denote negative electrostatic potential regions, blue areas correspond to positive electrostatic potential regions, and white areas represent neutral zones. The scale bar beneath indicates the corresponding electrostatic potential range (kT*e^-1^).

Since the cDNA sequence of *SjHAT1* in the NCBI database is predicted and incomplete, we utilized the rapid amplification of cDNA ends (RACE) technique to obtain its full-length cDNA sequence for further characterization. The full-length cDNA sequence of *SjHAT1* was successfully obtained through RACE assay, revealing a 1302 bp open reading frame (ORF) encoding 433 amino acids (NCBI accession no. PQ778043). Using the InterPro database, we identified that *Sj*HAT1 contains an N-terminal HAT1 domain (IPR019467, 14–175aa) and a C-terminal HAT type B catalytic subunit domain (IPR048776, 316–339aa). The N-terminal HAT1 domain catalyzes the transfer of an acetyl group from acetyl-CoA to lysine residues at the histone N-terminus [20]. By contrast, the C-terminal domain is not conserved and is dispensable for HAT1 catalytic activity [21]. To investigate the enzymatic function of *Sj*HAT1, we successfully expressed recombinant *Sj*HAT1 (r*Sj*HAT1) in *E. coli* and demonstrated its robust histone acetyltransferase activity through *in vitro* assays (Table 1). Notably, the enzymatic activity of r*Sj*HAT1 was dose-dependently inhibited by the compound DW-3–15, with complete inhibition at a concentration of 50 μM (Table 1). Meanwhile, DW-3–15 had no effect on the enzymatic activity of *Homo sapiens* rHAT1 (S1 Table). To validate whether *Sj*HAT1 has potential as a drug target, we performed a multiple sequence alignment of *Sj*HAT1 with HAT1 homologs from *Homo sapiens*, *Mus musculus*, *Rattus norvegicus*, *Schistosoma mansoni*, *Schistosoma haematobium*, *Clonorchis sinensis*, and *Fasciola hepatica*. Multi-sequence alignment revealed that *Sj*HAT1 shares approximately 34% sequence identity with the HAT1 proteins of human, mouse, and rat, and the key amino acids required for HAT1 enzymatic activity are highly conserved (Fig 1A). Phylogenetic analysis demonstrates that *Sj*HAT1 clusters in an evolutionarily distinct clade, and displays significantly higher sequence homology with HAT1 orthologs from other trematode species compared to non-parasitic flatworms, underscoring strong evolutionary conservation within parasitic flatworms (Fig 1B). Structural characterization of motif architecture further indicates that critical functional domains exhibit remarkable conservation across taxa (Fig 1B). Evolutionarily conserved protein core domains maintain functional integrity, while different spatial conformations facilitate functional specialization. Therefore, we employ AlphaFold3 to predict the tertiary structure of *Sj*HAT1 (S1 Fig and S2 Table) and conduct a comparative analysis with its human homolog *Hs*HAT1 (PDB: 2P0W). Structural superposition reveals that *Sj*HAT1 and *Hs*HAT1 share a conserved fold architecture, as evidenced by their significant global structural similarity (TM-score = 0.92), while exhibiting notable local divergence (RMSD = 2.40 Å). Molecular docking results reveal that among the top 100 docking conformations, the *Sj*HAT1-DW-3–15 complex demonstrates heterogeneous binding modes, whereas the *Hs*HAT1-DW-3–15 complex exhibits limited conformational diversity (Fig 1C). Furthermore, residue-level interaction analysis identifies distinct binding patterns. In the optimal binding model, we selected amino acids within 4 Å of the DW-3–15. *Sj*HAT1 engages the ligand primarily through Trp-187, Tyr-215, Phe-217, Tyr-218, Tyr-220, Arg-227, and Glu-266 (Fig 1D), whereas *Hs*HAT1 employs Gly-230, Gln-231, Ala-235, Glu-239, Lys-265, Leu-266, Asp-268, Phe-269, and Ile-315 (Fig 1E). Electrostatic potential mapping shows a significantly more electronegative binding cavity in *Sj*HAT1 compared to *Hs*HAT1. This charge disparity likely mediates divergent ligand-binding thermodynamics through modulation of ligand orientation and binding free energy (Fig 1F). These distinctive molecular characteristics, particularly the structural divergence from *Hs*HAT1, establish *Sj*HAT1 as a promising druggable target for schistosomiasis. Such divergence suggests that species-specific targeting of *Sj*HAT1 could mitigate host toxicity by avoiding interference with the host’s HAT1.

**Table 1:** Effect of DW-3-15 on the enzymatic activity of recombinant *Sj*HAT1.

Group	ΔRFU	ΔT (min)	CoA^**^ production(pmol)	Acetyltransferase (HAT) activity (μU/mL)^***^
Background	0	55	0	0
Positive control	235353	55	493	3
r*Sj*HAT1^*^ (2.5μL)	96077	55	178	130
r*Sj*HAT1 (2.5μL)+0.5 μM DW-3–15	30087	55	29	21
r*Sj*HAT1 (2.5μL)+5 μM DW-3–15	21095	55	9	6
r*Sj*HAT1 (2.5μL)+50 μM DW-3–15	11492	55	0	0

*The initial concentration of r*Sj*HAT1 was 2.03 mg/mL. After a 100-fold dilution, the addition of 2.5 μl resulted in an r*Sj*HAT1 content of 50 ng.

**The regression equation is Y = 442.58X + 17262, R^2^ = 0.99953.

***HAT activity (µU/mL) = [B/(∆T × V)] × D. B = Amount of CoA from standard curve (pmol). ΔRFU was used to to obtain B pmol of CoA generated by histone acetyltransferase during the reaction time. ΔT = reaction time (min). V = Original sample volume added into the reaction well (µL). D = Sample dilution factor. The raw data is shown in supporting information file [Table A in S1 Data].

### *SjHAT1* is highly expressed in the vitellaria of adult female worms

**Fig 2:**
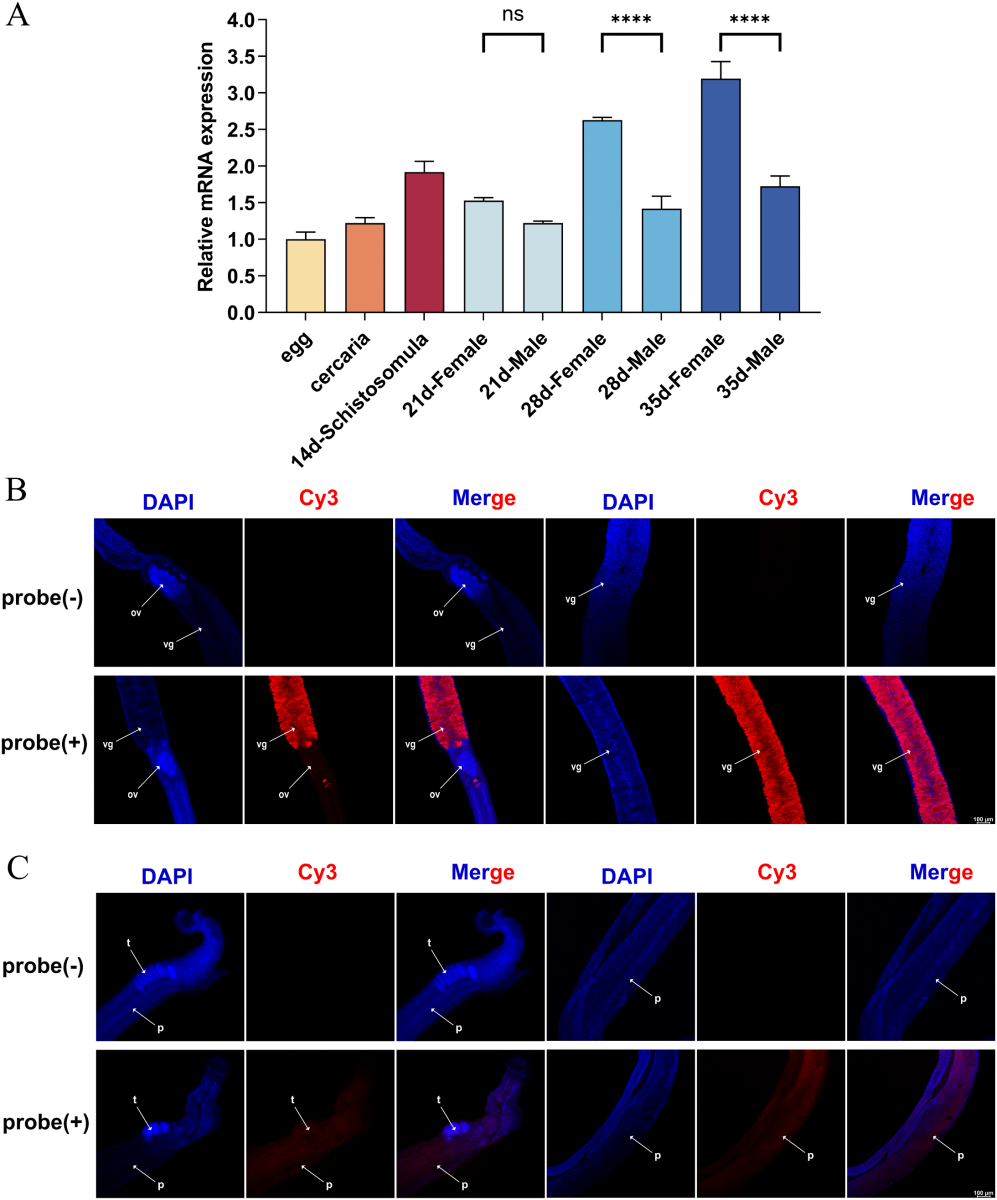
Expression and location of *Sj*HAT1 in adult worms of *S. japonicum.* **(A)** Transcriptional levels of *SjHAT1* in different developmental stages and genders of *S. japonicum*. N = 3 biological replicates, Student’s *t*-test, standard deviation (SD) is shown in the error bars. ‘ns’ indicates no significant difference (*p* > 0.05), ****p < 0.0001. The raw data is shown in supporting information file [Table B in S1 Data]. **(B)** Localization of *SjHAT1* in female *S. japonicum*. ‘ov’, ovary; ‘vg’, vitelline gland. Scale bars: 200 μm. **(C)** Localization of *SjHAT1* in male *S. japonicum*. ‘t’, testis; ‘p’, parenchymal cell. Scale bars: 200 μm.

To further characterize *Sj*HAT1 and facilitate future investigations into its physiological functions, the mRNA expression profiles of the *SjHAT1* across six critical developmental stages, including eggs, cercariae, 14-day-old juveniles, 21-, 28- and 35-day-old adult male/female worms were quantified. The results revealed that *SjHAT1* transcripts, while ubiquitously expressed, exhibited significant stage specificity and sexual dimorphism. The highest expression level of *SjHAT1* was observed in 35-day-old females, whereas the lowest expression was detected in eggs. Notably, sex-specific differences became pronounced in 28- and 35-day-old adults, with *SjHAT1* expression in females significantly exceeding that in males (*p* < 0.0001, Fig 2A). This differential expression pattern suggests that *SjHAT1* may play a pivotal role in sexual maturation and female reproductive development in schistosomes.

Subsequently, we investigated the tissue-specific localization of *SjHAT1* in 35-day-old adult worms. Fluorescence *in situ* hybridization (FISH) revealed that *SjHAT1* is predominantly expressed in the vitellaria of female worms (Fig 2B) and in the parenchymal cells adjacent to the gynecophoral canal of male worms (Fig 2C). The female-biased expression pattern in vitellaria, a specialized organ responsible for vitellocytes production [22], further implicates *Sj*HAT1 in reproductive regulation.

### *SjHAT1* knockdown impairs viability and oviposition of adult *S. japonicum in vitro*

**Fig 3:**
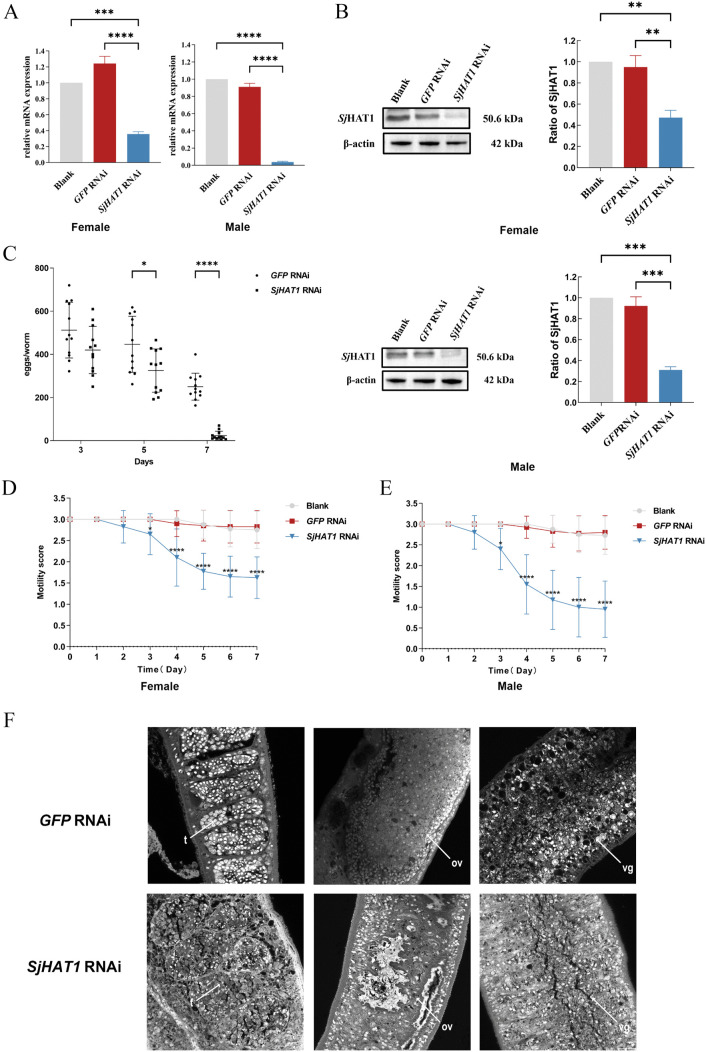
*SjHAT1* knockdown disrupts the viability and oviposition in adult worms *in vitro.* **(A)** Changes in *SjHAT1* transcript level after *SjHAT1*-dsRNA treatment. Standard deviation (SD) is shown in the error bars, n = 3 bilogical replicates, Student’s *t*-test, ***p < 0.001, ****p < 0.0001. **(B)** Changes in *Sj*HAT1 protein expression after *SjHAT1*-dsRNA treatment. Standard deviation (SD) is shown in the error bars, n = 3 biological replicates, **p < 0.01. **(C)** Altered oviposition in females after *SjHAT1*-dsRNA treatment. Error bars demonstrate standard deviation (SD), n = 12, Two-Way ANOVA, *p < 0.05, ****p < 0.0001. The raw data is shown in supporting information file [Table C in S1 Data]. **(D)** Altered Motility in females after *SjHAT1*-dsRNA treatment. Error bars represent standard deviation (SD), n = 40, Two-Way ANOVA, *p < 0.05, ****p < 0.0001. **(E)** Altered Motility in males after *SjHAT1*-dsRNA treatment (n = 40), Two-Way ANOVA, *p < 0.05, ****p < 0.0001. The raw data is shown in supporting information file [Table D in S1 Data]. **(F)** Morphological changes in the reproductive organs of worms after *SjHAT1*-dsRNA treatment. ‘t’, testis; ‘ov’, ovary; ‘vg’, vitelline gland. Scale bars: 20 μm.

To investigate the functions of *Sj*HAT1, we conducted RNA interference (RNAi) in paired adult worms *in vitro*. We designed two dsRNAs, *SjHAT1*-dsRNA1 and *SjHAT1*-dsRNA2, targeting the *N*-terminal and *C*-terminal domains of *Sj*HAT1, respectively. Through preliminary experiments comparing the interference efficiency of *SjHAT1*-dsRNA1 (30 μg/mL), *SjHAT1*-dsRNA2 (30 μg/mL), and *SjHAT1*-dsRNA1/2 (15 μg/mL *SjHAT1*-dsRNA1 and 15 μg/mL *SjHAT1*-dsRNA2), we found that the *SjHAT1*-dsRNA1/2 exhibited the highest interference efficiency (S2 Fig). Hence, in the following studies, *SjHAT1*-dsRNA1/2 was added on day 1, day 3, and day 7 using the soaking method [3]. The results showed that *in vitro* RNAi significantly reduced both the transcriptional level and protein expression of the *Sj*HAT1 in female and male worms (Fig 3A and 3B).

After 7 days of *SjHAT1*-dsRNA treatment, *S. japonicum* adult worms exhibited pronounced phenotypic alterations, including markedly reduced egg laying by females and compromised locomotor activity. As shown in Fig 3C, female oviposition was significantly reduced at day 5 posttreatment with *SjHAT1*-dsRNA interference (*p* < 0.05), reaching the lowest at day 7 (*p* < 0.0001). The reduction rate of female oviposition was 90.8% after 7 days interference. Similarly, both female and male worms exhibited significantly lower viability compared with the control group from day 3 onward (Fig 3D and 3E, *p* < 0.05), with scores reaching their lowest levels by 7 days posttreatment (Fig 3D and 3E, *p* < 0.0001). At day 7, the intervention showed no statistically significant impact on worm pairing (S3 Fig).

To assess reproductive organ development following *SjHAT1* knockdown, worms were carmine-stained and examined by confocal laser scanning microscopy (CLSM). We found that knockdown of *SjHAT*1 severely disrupted the morphology of the reproductive organs in both sexes of *S. japonicum*. In males, the testes became disorganized, with indistinct lobular boundaries, loose stromal matrices, reduced spermatogonial populations, and abnormal cellular morphology. In females, the vitelline glands were atrophic, characterized by effaced lobular boundaries, fewer mature vitelline cells, and markedly shrunken ovaries (Fig 3F).

**Fig 4:**
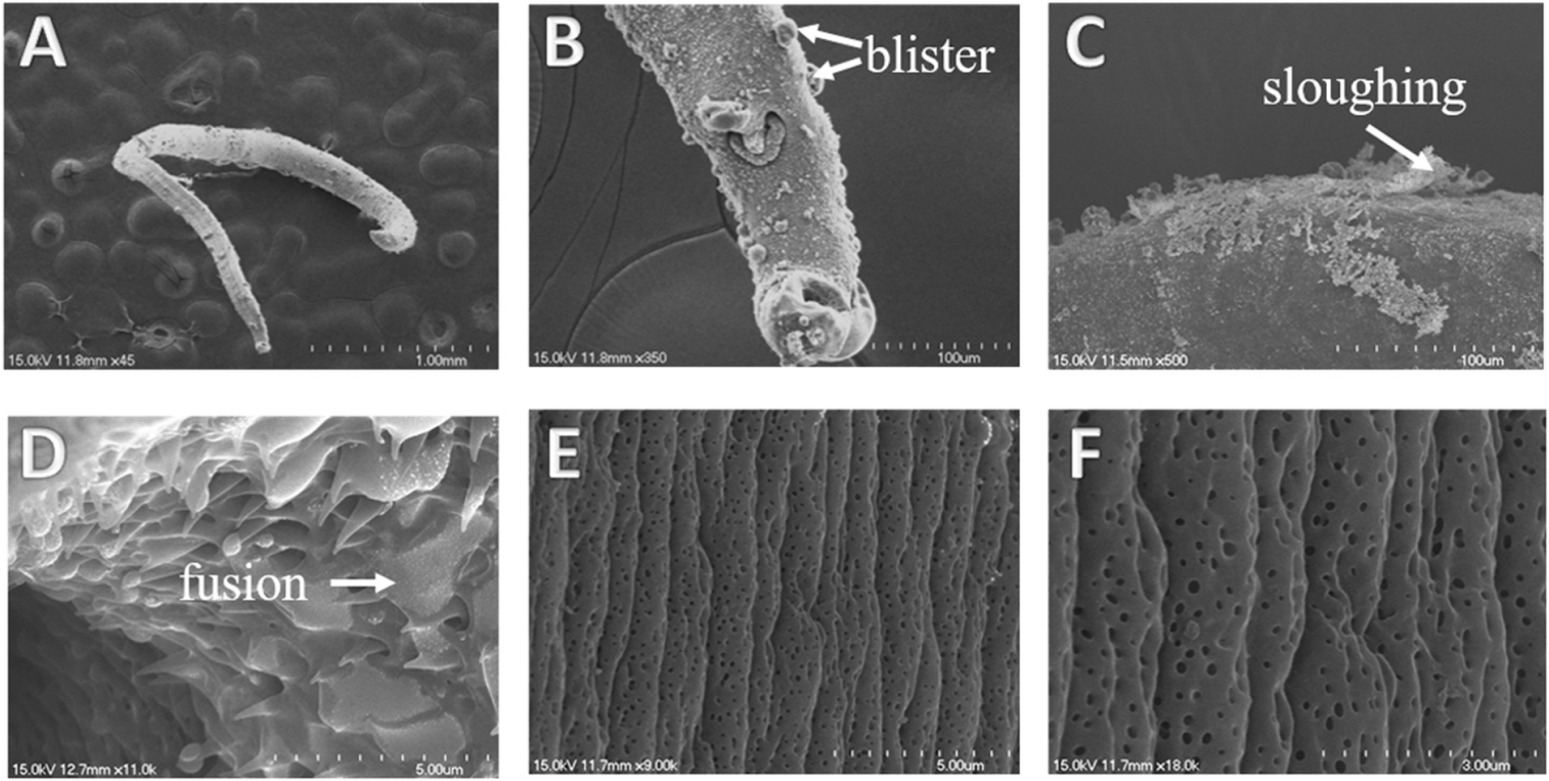
Scanning electron micrographs of the tegument of female *S. japonicum* worms after *SjHAT1* RNAi for 3 days. **(A)** The whole worm is swollen and shrunken; **(B)** mild swelling accompanied by numerous blisters is observed at the anterior end of female worm; **(C)** extensive sloughing is observed on the tegument in the mid-body of female worm; (D) fusion of the spines in the ventral sucker are obvious; **(E)**-**(F)** no significant changes are observed in the transverse ridge-like structures on the tegument of female worm. The ridges arranged orderly accompanied by uniformly distributed pores. Scalebars: A: 1 mm; B: 100 μm; C: 100 μm; D: 5 μm; E: 5 μm; F: 3 μm.

It is well known that an intact tegument is crucial for worm survival [7]. Disruption of the tegument leads to exposure of the worm’s antigens, rendering it more susceptible to the host immune system [23,24]. We therefore employed scanning electron microscopy (SEM) to examine the tegumental changes after *SjHAT*1 knockdown. According to the *in vitro* experimental results, worm viability began to decline significantly by 3 days post-interference. Consequently, parasites collected on the third day after interference were subjected to SEM observation. Under SEM, the tegument of the female worm in the control group remained intact, with spines uniformly arranged in the ventral sucker (S4 Fig). In contrast, following *SjHAT1*-dsRNA intervention for 3 days, distinct tegumental damage was evident. The anterior region of female worms exhibited mild swelling accompanied by numerous blisters (Fig 4B). The mid-body tegument displayed extensive sloughing (Fig 4C), and obvious fusion of the spines occurred in the ventral suck of females (Fig 4D). Silencing *SjHAT1* induces morphological changes (Fig 4) similar to those observed after 72 h treatment with 100 μM DW-3–15, although the severity of damage is less pronounced comparable to the DW-3–15 treatment group (S5 Fig). These *in vitro* results indicate that *SjHAT1* knockdown critically impairs survival and reproductive functions in adult *S. japonicum*.

### *SjHAT1* is essential for the survival, pairing and oviposition of *S. japonicum in vivo*

Our *in vitro* results indicate *Sj*HAT1 is required for maintaining the adult worm viability and is crucial for reproductive processes in *S. japonicum*. However, the *in vitro SjHAT1*-dsRNA interference experiment was conducted on paired adult worms and did not address the role of *Sj*HAT1 at other developmental stages. Furthermore, it is technically challenging to collect and culture schistosomula of different developmental stages *in vitro*. Thus, we carried out *in vivo* RNAi experiments to assess the effects of *SjHAT1* knockdown across parasite developmental stages. Mice infected with *S. japonicum* cercariae received a tail vein injection of 10 μg *SjHAT1*-dsRNA at 14 days post-infection (dpi), followed by repeated injections of 10 μg dsRNA at 18, 22, 26, and 30 dpi. On day 35 post-infection, the parasites were harvest through perfusion of the hepatic portal system and mesenteric veins, and mouse livers were collected for egg counting and histopathological analysis (H&E staining). Worm burden reduction and hepatic egg burden reduction were calculated.

**Fig 5:**
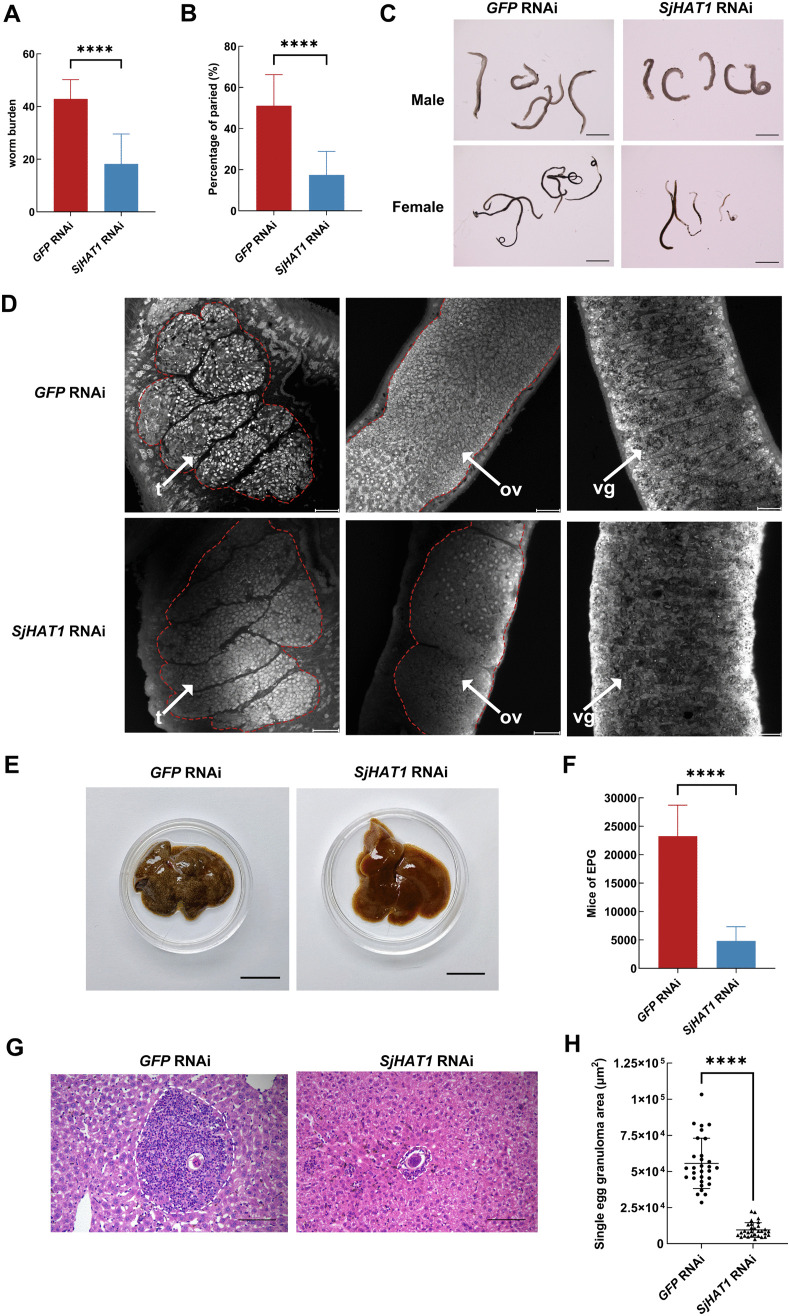
*Sj*HAT1 affects survival, pairing and oviposition of *S. japonicum in vivo.* On the 14^th^, 18^th^, 22^th^, 26^th^ and 30^th^ days postinfection, *SjHAT1*-dsRNA was injected through the tail vein, and the hepatic portal vein was perfused on the 35^th^ day. **(A)** Worm burden of the parasites recovered at 35 dpi in *GFP* control RNAi and *SjHAT1* RNAi groups. N = 3 biological replicates, 3 mice of each biological replicates. Student’s *t*-test, ****p < 0.0001. The raw data is shown in supporting information file [Table E in S1 Data]. **(B)** Comparison of the pairing rates of recovered worms between *GFP* and *SjHAT1* dsRNA groups. N = 3 biological replicate, 3 mice of each biological replicates. Student’s *t*-*t*est, ****p < 0.0001. The raw data is shown in supporting information file [Table F in S1 Data]. **(C)** Morphological observation of worms after RNAi *in vivo*. Scale bars: 2000 μm. **(D)** Morphological changes in the reproductive organs of worms after RNAi *in vivo*. ‘t’, testis; ‘ov’, ovary; ‘vg’, vitelline gland. Scale bars: 20 μm. **(E)** Gross observations of the mouse liver in the *GFP* and *SjHAT1* dsRNA groups. Scale bars: 1 cm. **(F)** Eggs per gram of the liver comparation between the *GFP* and *SjHAT1* dsRNA groups (n = 90). Student’s *t*-tes*t*, ****p < 0.0001. The raw data is shown in supporting information file [Table G in S1 Data]. **(G)** Histological assessment of mouse liver by H&E staining. Scale bars: 100 μm. **(H)** Statistical analysis of the size of egg granuloma area after RNAi *in vivo* (n = 30)*.* Student’s *t*-test, ****p < 0.001. Error bars re*p*resen*t* standard deviation (SD). The raw data is shown in supporting information file [Table H in S1 Data].

In these experiments, mice that received control *GFP*-dsRNA showed no significant differences in worm or egg burdens compared to blank controls injected with saline (S6A, S6B and S6E Fig). Parasites recovered from *GFP*-dsRNA control mice were morphologically normal (S6C and S6D Fig), and there were no significant differences in the number or size of liver egg granulomas between *GFP*-dsRNA and blank control groups (S6F and S6G Fig). These findings confirm that *GFP*-dsRNA had no appreciable effect on the parasites. In contrast, mice treated with *SjHAT1*-dsRNA showed a 57.5% reduction in worm burden (Fig 5A, *p* < 0.0001) and a 65.9% reduction in pairing (Fig 5B, *p* < 0.0001) compared to the *GFP*-dsRNA controls. Moreover, many female worms from the *SjHAT1* knockdown group exhibited developmental arrest (Fig 5C), with 32.9% remaining immature, which was significantly higher than that the 18.9% observed in the *GFP* control group [*χ*^2^(1) =5.528, *p* = 0.0187, S3 Table]. These results indicate that the downregulation of *SjHAT1* impairs the development and sexual maturity of female worms, thereby reducing their egg-laying capacity. Consistent with these observations, CLSM analysis revealed severe testicular hypoplasia in males, while females showed ovarian degeneration and vitelline gland atrophy characterized by diminished lobular boundaries and reduced mature vitellocyte count (Fig 5D). Eggs are the key factor responsible for the pathogenesis of schistosomiasis [25–28]. The formation of hepatic egg granulomas depends not only on the number of deposited eggs but also on their viabilities. Pathologically, *SjHAT1*-RNAi livers displayed significantly attenuated hepatic granuloma formation (Fig 5E) and 79.2% reduction in egg deposition (*p* < 0.001, Fig 5F). H&E staining confirmed 82.9% smaller granuloma areas 9480 ± 5029 μm^2^ vs control 55542 ± 17398 μm^2^ (*p* < 0.001, Fig 5G and 5H), demonstrating impaired oviposition capacity post-RNAi. These findings indicate that *SjHAT1* knockdown *in vivo* severely impairs oviposition and significantly mitigates egg-induced hepatic pathology.

### *SjHAT*1 knockdown modulates ABC transporter pathway in females and tyrosine metabolism in males

**Fig 6:**
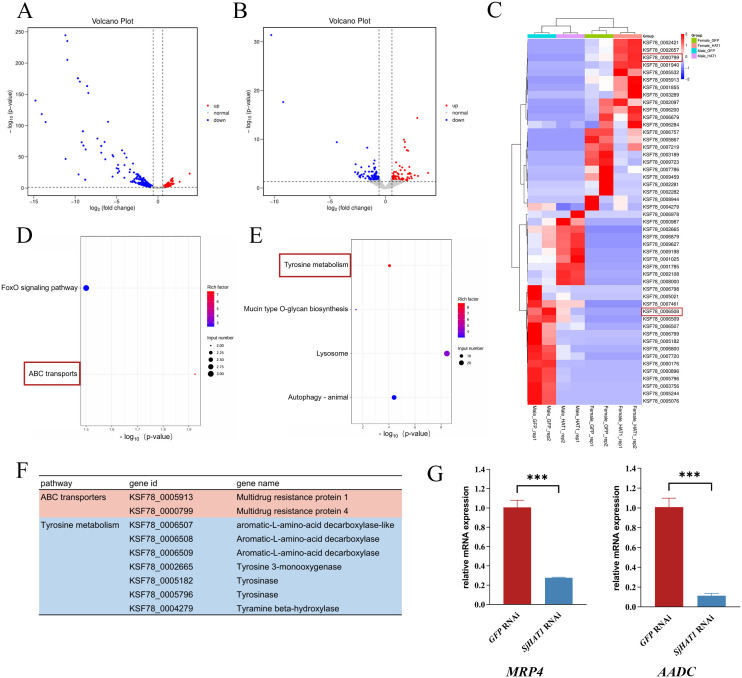
*Sj*HAT1 regulates different pathways in female and male worms. **(A)** Volcano plot illustrating differentially expressed genes (DEGs) in female parasites. **(B)** Volcano plot illustrating DEGs in male worms. DEGs were defined as |log2 Fold Change| ≥ 0.58 and *p*-value< 0.05. **(C)** Heatmap of the differentially expressed genes. **(D)** KEGG pathway enrichment analysis of DEGs in female worms. **(E)** KEGG pathway enrichment analysis of DEGs in male worms. **(F)** Differentially expressed genes following *SjHAT1* knockdown. (G) qRT-PCR validation of *MRP4* (female) and *AADC* (male) expression. N = 3 bilogical replicates, Student’s *t*-*t*est, ***p < 0.001. Error bars represent standard deviation (SD).

Having demonstrated that *SjHAT1* knockdown disrupts the normal morphology, growth, development and oviposition of *S. japonicum*, we next investigated the molecular pathways regulated by *SjHAT1*. We performed RNA sequencing (RNA-seq) analysis on male and female worms following *SjHAT1* knockdown *in vitro*. The RNA-seq results revealed a total of 206 differentially expressed genes (DEGs) in female worms (86 upregulated and 120 downregulated; Fig 6A) and 786 DEGs in male worms (242 upregulated and 584 downregulated; Fig 6B). Gene Ontology (GO) analysis and KEGG pathway enrichment were then applied to these DEGs.

After the knockdown of *SjHAT1*, genes with the most signiﬁcant expression changes included *copine-9* (KSF78_0001940), *calcium activated potassium channel subunit* (KSF78_0002097), *tyrosine-protein kinase FRK* (KSF78_0003289), *ribonuclease T2* (KSF78_0002281), *multidrug resistance-associated protein 4* (*MRP4*, KSF78_0000799) and *multidrug resistance protein 1* (KSF78_0005913) in females, and *neuronal acetylcholine receptor subunit alpha-2* (KSF78_0006978), *aromatic L-amino acid decarboxylase* (*AADC*, KSF78_0006508), *tyrosinase* (KSF78_0005796, KSF78_0005182), *tyrosine hydroxylase* (KSF78_0002665) in males (Fig 6C and 6F).

In female worms, the downregulated DEGs were significantly enriched in the ATP binding cassette (ABC) transporter pathway (Fig 6D), with *MRP4* being specifically and significantly downregulated (*p* = 0.01931655). Consistently, GO analysis linked this female DEG to transmembrane transport processes (GO:0055085). In male worms, the downregulated DEGs were predominantly enriched in the tyrosine metabolism pathway (Fig 6E). Notably, *AADC*, a gene normally highly expressed in males, was dramatically downregulated (*p* = 5.49458E-07). GO analysis indicated that this enzyme is involved in carboxylic acid metabolic processes (GO:0019752). Finally, qRT-PCR validation confirmed that *SjHAT1* knockdown significantly reduced *MRP4* expression in female worms and *AADC* transcriptional levels in male worms (Fig 6G), consistent with the RNA-seq data.

## Discussion

In our previous work, we observed that the praziquantel derivative DW-3–15, has exceptional schistosomicidal activity against all developmental stages of *S. japonicum*, with >60% worm reduction against juveniles [10]. This broad efficacy contrasts sharply with PZQ, which mainly targets adult worms [29]. Notably, DW-3–15 was found to markedly suppress the expression of *Sj*HAT1 in adult worms. Similarly, curcumin, a histone acetyltransferase inhibitor that also suppresses *Sj*HAT1 expression, elicited phenotypic changes resembling those caused by DW-3–15 [13]. These findings suggest that *Sj*HAT1 is a critical molecular target underlying the anti-schistosomal effects of DW-3–15. However, the existing *SjHAT1* cDNA sequence in the NCBI database is incomplete and based on predictions. We used RACE to clone the full-length *SjHAT1* cDNA (NCBI accession no. PQ778043) and characterized the encoded protein. We identified four nucleotide differences from the predicted sequence, including a nonsynonymous mutation (cysteine to serine) at residue 311. Phylogenetic analysis reveals that *Sj*HAT1 forms a distinct clade separate from humans and other mammals, whereas it exhibits remarkable evolutionary conservation across trematode species (Fig 1B). Molecular docking analysis demonstrates that both the critical residues mediating the *Sj*HAT1-DW-3–15 interaction and the surface electrostatic potential distribution showed significant divergence from corresponding features in *Hs*HAT1 (Fig 1C–1F). This divergency implies that pharmacological agents could be designed to specifically target *Sj*HAT1’s unique domains, potentially minimizing off-target effects on homologous host proteins and thereby mitigating toxicity. Importantly, our qPCR analysis confirmed constitutive expression of *SjHAT1* across all developmental stages and both sexes of *S. japonicum* (Fig 2A), indicating its essential biological role throughout the parasite’s life cycle. Notably, recombinant *Sj*HAT1 exhibited robust histone acetyltransferase activity, which was inhibited by the compound DW-3–15 in a dose-dependent manner. This pharmacologic property suggests a potential mechanism for the schistosomicidal effects of DW-3–15 against multiple developmental stages of *S. japonicum* [9–12]. Furthermore, the expression level of *SjHAT1* gradually increased as the parasite matured, with significantly higher expression observed in mature female worms compared to their male counterparts of the same age. This sex-biased expression pattern suggests that *Sj*HAT1 may play a critical role in the development and maturity of the female worms. Consistent with the expression data, FISH revealed that *SjHAT1* is predominantly localized in the vitellaria of female *S. japonicum* (Fig 2B). The vitellaria occupy approximately two-thirds of the female body and comprise numerous transversely arranged vitelline lobules extending dorsoventrally. These lobules contain vitelline cells at various developmental stages, which provide both nutrients for fertilized ova and polyphenolic proteins critical for eggshell formation [22,28,30,31]. Mature female schistosomes produce an average of 3,500 eggs per day. Each mature egg consists of one fertilized ovum surrounded by approximately 20 vitelline cells, requiring the female to synthesize 70,000 vitelline cells daily (equivalent to 50 cells per minute) to sustain oviposition [30]. Disruption of vitelline gland function would directly impair egg production. Targeting egg-laying mechanisms not only mitigates immunopathological damage in the host but also represents a strategic intervention for controlling schistosomiasis transmission [28].

Our *in vitro* experiments further reinforce the potential of *Sj*HAT1 as a promising druggable target for schistosomiasis. Specifically, dsRNA-mediated silencing of *SjHAT1* significantly reduced schistosome viability and impaired egg-laying capacity in females, exhibiting time-dependent inhibitory effects. Morphological analysis indicated that *SjHAT1*-dsRNA impairs the integrity of the female tegument (Fig 4C), a structure critical for schistosome survival [7,23,24]. Furthermore, the tegumental changes observed after *SjHAT1* RNA interference closely resembled those induced by DW-3–15. In addition, *SjHAT1* knockdown induced notable structural changes in the reproductive organs of *S. japonicum* adult worms. Following RNA interference, female ovaries displayed severe atrophy and distinct morphological abnormalities, including a marked reduction in mature oocytes, disappearance of intercellular spaces, disorganized cell arrangement, and vacuole formation (Fig 3F). These phenotypic alterations were consistent with those induced by the HAT inhibitor PU139 [18]. A previous study also demonstrated that HAT1 was highly expressed in ovarian granulosa cells (GCs) from young mice and its expression was significantly decreased in aged GCs. siRNA mediated inhibition of *HAT1* in GCs decreased the polar body extrusion rate, and increased meiotic defects and aneuploidy in oocytes [32]. Together, these results illustrate the essential role of *Sj*HAT1 in regulating reproductive organ development and egg-laying processes in schistosomes.

Although our *in vitro* results demonstrated significant anti-schistosomal activity via *SjHAT1* knockdown, it is generally acknowledged that this observed efficacy *in vitro* does not always translate in corresponding parasiticidal effects *in vivo* [33]. In our case, however, *SjHAT1* RNAi *in vivo* caused a 57.5% reduction (*p* < 0.0001) in worm burden (Fig 5A) and 65.9% reduction (*p* < 0.0001) in pairing rate (Fig 5B). Furthermore, the intervention markedly inhibited the developmental progression and maturation of female worms (Fig 5C), with 32.9% arrested at the juvenile stage. This developmental impairment consequently led to compromised oviposition capacity in females. The liver egg deposition was reduced by 79.2% after interference of *SjHAT1*, which in turn signiﬁcantly attenuated the pathological damage to the mice (Fig 5F–5H). It is well-known that schistosomes are dioecious, with separate male and female sexes [34]. The sexual development of female schistosomes is entirely dependent on pairing with a male schistosome [35]. In the absence of males, the ovaries and vitellaria of females remain in a primordial state, rendering immature females incapable of laying eggs [36]. Further, it has been shown that physical contact with a male worm, and not insemination, is sufficient to induce female development and the production of viable eggs [37]. Chen et al [38] identified that β-alanyl-tryptamine (BATT), a non-ribosomal peptide produced by male schistosomes is responsible for inducing female sexual development and egg reproduction. Crucially, exogenous BATT alone is sufficient to induce egg laying in immature female parasites [38]. Thus, the reduced male-female interaction, primarily resulting from the decreased male worm population following *SjHAT1* knockdown, leads to delayed development of female worms. Additionally, functionally impaired male worms exhibit deficient BATT synthesis, which is essential for triggering maturation and oviposition in females. Moreover, similar phenotypic impairments, including testicular hypoplasia, ovarian degeneration and vitellaria atrophy, were observed in reproductive organs of worms recovered from *SjHAT1* RNAi-treated mice *in vivo* (Fig 5D). These *in vivo* results demonstrate that *Sj*HAT1 critically regulates the growth, pairing and oviposition in *S. japonicum*, suggesting that *Sj*HAT1 is a novel druggable target.

Another striking result from our RNA-seq data is the signiﬁcant downregulation of *multidrug resistance-associated protein 4* (*MRP4*, KSF78_0000799) in females and *aromatic L-amino acid decarboxylase* (*AADC*, KSF78_0006508) in males. AADCs are the enzymes catalyzing the decarboxylation of aromatic amino acids (such as tryptophan and tyrosine) to generate monoamines (such as tryptamine and dopamine), which play diverse physiological and biosynthetic roles in living organisms [39]. It has been reported that in planarians, AADC collaborates with nonribosomal peptide synthetase (NRPS) to catalyze the synthesis of BATT [40]. BATT is an essential pheromone that stimulates the maturation of the female reproductive system and promotes egg-laying. Furthermore, *in vitro* experiments demonstrate that exogenous BATT can replace male worms to induce female reproductive system development and egg production [38]. Knockdown of *AADC* in sexual planarians resulted in ovary ablation and loss of accessory reproductive organs, including vitellaria, oviducts, sperm ducts, and gonopore [41]. Previous studies have found that *AADC* is specifically highly expressed in male *S. japonicum* and is predominantly localized on the inner surface of the gynecophoric canal [42], which exhibits spatial overlap with the distribution of *SjHAT1* in male worms. Therefore, we have reason to infer that targeted downregulation of *SjHAT1* suppresses the expression of *AADC*, thereby blocking the synthesis and secretion of the key pheromone BATT in male worms, thus inhibiting the development of the female reproductive system and oviposition. However, how is the BATT signal transduced to female worms? Existing studies have shown that signaling molecules such as linoleic acid, epidermal growth factor, and insulin-like growth factor can enter schistosomes through their excretory system, activating internal signaling pathways [43]. ATP binding cassette (ABC) transporters are the main components of the excretory system. Multidrug resistance-associated proteins (MRPs) are members of ABC transporters. Knockdown of *Sm*MRP1 in *S. mansoni* adults disrupts egg production and mitigates liver pathology [44]. Our RNA-seq data also revealed a significant downregulation of *multidrug resistance-associated protein 4* (*MRP4*) in female worms, indicating that BATT pheromone might be transduced to females through the MRPs. Taken together, we hypothesize that downregulation of *SjHAT1* inhibits the expression of key enzymes such as *AADC* in male worms, blocking the synthesis and excretion of BATT signals in males; by suppressing the ABC transporter system in female worms, it further hinders BATT signal transduction, thereby suppressing the development of the female reproductive system and oviposition. Verification of this hypothesis could provide new strategies for the prevention and control of schistosomiasis, as well as novel targets for the development of anti-schistosomal drugs.

In conclusion, we have characterized *Sj*HAT1 as a novel anti-schistosomal drug target. It is predominantly expressed in the vitellaria of female worms and in the parenchyma adjacent to the gynecophoral canal of male worms. Knockdown of *SjHAT1* impairs worm viability, development and reproduction both *in vitro* and *in vivo*. Furthermore, we hypothesize that dual-sex regulatory mechanisms may play a key role in the process of schistosome female egg-laying. Developing specific *Sj*HAT1 inhibitors could yield novel compounds to control schistosomiasis. Future studies on the downstream mechanisms of *Sj*HAT1 will improve our understanding of sexual maturation and oviposition of schistosomes.

## Methods

### Ethics

All animal experiments were conducted in strict accordance with the recommendations in the Guide for the Care and Use of Laboratory Animals of the National Institutes of Health. All efforts were made to relieve the suffering of the experimental animals. The protocol (including mortality aspects) was approved by the Institutional Animal Care and Use Committee (IACUC) of Soochow University (Permit Number: 2007–13).

### Animals and parasites

Female C57BL/6 mice (6–8 weeks old, 20.0 ± 5 g) were provided by the Center for Experimental Animals at Soochow University (Suzhou, China). All mice were raised under specific pathogen-free (SPF) conditions with a controlled temperature of 22°C, humidity of 55%, and photoperiod (12 h light, 12 h dark). Each mouse was transcutaneously infected with 60 ± 2 *Schistosoma japonicum* cercariae (Chinese mainland strain) shedding from snails (*Oncomelania hupensis*), which were provided by the Institute of Schistosomiasis Control in Jiangsu Province (Wuxi, China). *S. japonicum* worms were recovered from the infected mice by perfusion through the hepatic portal vein [45]. Three worm pairs were cultured at 37˚C in 5% CO_2_ in 1 ml m-AB169 medium in a 24-well plate [46]. The medium was changed every other day.

### Rapid amplification of cDNA ends (RACE)

Based on the predicted sequences (GenBank: FN318824.1) of *SjHAT1* in the NCBI database, an intermediate fragment was amplified by PCR. Subsequently, gene-specific primers (GSP) and nested gene-specific primers (NGSP) were designed from the intermediate fragment by Primer Premier 6 (S4 Table). RACE was performed with SMARTer RACE cDNA Amplification Kit (Clontech, USA) to obtain the terminal sequences. The purified amplification products were directionally cloned into the pRACE vector using the In-Fusion Cloning System. The resultant recombinant plasmids were subjected to bidirectional Sanger sequencing with M13 universal primers, and the obtained sequences were computationally assembled to determine the full-length cDNA of *SjHAT1*. The open reading frame (ORF) region was analyzed using the ORF Finder tool in the NCBI database, yielding a single complete ORF and its corresponding amino acid sequence.

### Multiple sequence alignment and phylogenetic analysis

Homologous proteins of HAT1 across species were retrieved from the UniProtKB/Swiss-Prot database. Multiple sequence alignment was performed with MAFFT v7.520. Putative conserved protein motifs were identified via MEME Suite. The phylogenetic tree construction via the Maximum Likelihood method in MEGA11 with 1000 bootstrap replicates for node support evaluation, and subsequently visualized using Chiplot (https://www.chiplot.online).

### Three-Dimensional structure of *Sj*HAT1 and molecular docking analysis with DW-3–15

The three-dimensional structure of *Sj*HAT1 was predicted using AlphaFold3 with default parameters, and the model with the highest confidence score was selected for further analysis [47]. Model reliability was evaluated using predicted local distance difference test (pLDDT), predicted TM-score (pTM), and the predicted aligned error (PAE) matrix. Structural validation was performed using MolProbity, including assessment of MolProbity score, clashscore, and side-chain conformations [48]. Additional validation was conducted using the SAVES v6.1 server, including Ramachandran plot, PROCHECK, ERRAT, and Verify3D analyses, to comprehensively assess the stereochemical quality and structural reliability of the model. The global structural alignment between *Sj*HAT1 and *Hs*HAT1 was performed using the TM-align algorithm under default parameters to calculate the TM-score. Additionally, the Combinatorial Extension (CE) algorithm implemented in PyMOL 3.2 was utilized for local structural superposition. The root-mean-square deviation (RMSD) of Cα atoms was calculated to assess regional conformational differences between the two proteins.

For the DW-3–15/*Hs*HAT1 and DW-3–15/*Sj*HAT1 complexes, a total of 1,000 structural models were generated per complex (25 MSAs × 20 random seeds × 2 samplings). These models were subsequently ranked using the interface-predicted TM-score (ipTM) metric, with the top-100-ranked model from each complex selected for further characterization. Structural predictions were conducted using EnsembleFold [49], a novel framework integrating the CASP15-winning DMFold algorithm [50] with an enhanced AlphaFold3 engine [47]. This integrative approach combines high-fidelity multiple sequence alignment MSA construction with advanced deep learning architectures, enabling accurate modeling of both monomeric and multimeric biomolecular complexes.

### Expression and purification of recombinant *Sj*HAT1 (r*Sj*HAT1) in *E. coli*

The open reading frame (ORF) of SjHAT1 was amplified with forward primer 5’- CGGAATTC ATGGACAGTCTGAATACCAGAA-3’ and reverse primer 5’- CCGCTCGAG TTAAGATTGTTTTTTAAGTGAAGAGACG-3’ using PCR Master Mix (Takara Bio, Shiga, Japan) and cDNA template prepared from adult worms. The PCR product was digested with EcoRI and XhoI (Takara Bio, Shiga, Japan) enzymes and gel-purified, then cloned into expression plasmid pGEX-4T-1. Positive clones were screened and confirmed by DNA sequencing. The restructured plasmid was transformed into Transetta (DE3) (TransGene Biotech Co., Ltd, Bejing, China), and the transformed cells were grown by shaking (220 rpm) in LB medium containing 100 μg/ml ampicillin. Recombinant *Sj*HAT1 was expressed as a glutathione-S-transferase-*Sj*HAT1 fusion protein induced by 0.5 mM isopropy1-β-d-thiogalactoside (IPTG) at 37˚C for 6 h. After induction, the cells were harvested by centrifugation at 5,000 g for 20 min at 4˚C. Add 10 mL pre-cooled PBS containing 1 × Protease inhibitor cocktail for bacterial cell extracts (Beyotime Biotech, Shanghai, China) to1 g of wet bacterial pellet. Vortex the mixture thoroughly to resuspend the cells. Sonicate the suspension on ice using the following parameters: 40% amplitude, 5 s pulses followed by 5 s intervals, for a total duration of 20 min. Centrifuge the lysate at 12,000 rpm for 20 min at 4°C, then collect the supernatant. The supernatant were purified by HyPur T GST 4FF PrePacked Gravity Column (Sangon Biotech, Shanghai, China). Then the protein concentration was determined by a Bradford Protein Assay kit (Beyotime Biotech, Shanghai, China).

### Determination of r*Sj*HAT1 enzymatic activity

The enzyme activity of r*Sj*HAT1 was assessed using Histone Acetyltransferase Activity Assay Kit (ab204709, Abcam, USA). Experiments were conducted in duplicate using black flat-bottomed 96-well microplate (Thermo Fisher, Shanghai, China). Standard Curve: standards and enzyme mixtures were prepared to generate standard curve. For each reaction, 50 µL of Histone Acetyltransferase Reaction Mix was prepared as follows: 30 µL HAT assay buffer, 4µL H3 peptide, 10 µL substrate mix, 2 µL Developer, 2 µL PicoProbe, 2 µL Acetyl CoA. Add 50 µL of the reaction mix to the standard (50 µL), sample (50 µL containing 50 ng r*Sj*HAT1), positive control (50 µL) and background (50 µL) wells, followed by incubation at 25°C for 60 minutes. Fluorescence intensity was recorded every 5 min at excitation/emission wavelengths of 535/587 nm. HAT activity was calculated as: HAT activity (µU/mL) = [B/(∆T × V)] × D. Where: B = Amount of CoA from Standard Curve (pmol). ΔT = Reaction time (min). V = Original sample volume added into the reaction well (µL). D = Sample dilution factor. To assess whether DW-3–15-mediated inhibition of *Sj*HAT1, reaction systems containing 50 ng r*Sj*HAT1 were supplemented with DW-3–15 at final concentrations of 0.5, 5, and 50 μM, respectively.

### Quantitative real-time PCR (qRT-PCR)

TRIzol Reagent (Invitrogen, USA) was used to extract total RNA from different developmental stages of *S. japonicum*, including eggs, cercariae, 14-day schistosomula, 21-day, 28-day, and 35-day adult male and female worms. Subsequently, 1 μg total RNA was used for first-strand cDNA synthesis using the RevertAid First-Strand cDNA Synthesis Kit (Thermo Fisher Scientific, USA). The cDNA was used as a template for qRT-PCR with ChamQ Universal SYBR qPCR Master Mix (Vazyme, China) and 0.4 µM forward and reverse primers. Amplification was performed on CFX96 Touch Quantitative Real-Time PCR System (Bio-Rad, USA) under standardized conditions: 95°C for 3 min, followed by 40 cycles of 95°C for 10 s, 60°C for 30 s, 72°C for 10 s; melt curve analysis: 65°C for 5 s, 95°C for 5 s. The qPCR primers (S4 Table) were designed with Primer Premier 6.0 software and validated through melting curve analysis. Relative quantification was performed using the 2^-ΔΔCt^ method with *S. japonicum* 26S proteasome non-ATPase regulatory subunit 4 (*SjPSMD4*, GenBank No. FN320595.1) as the internal reference.

### Fluorescence *in situ* hybridization (FISH)

Males and females recovered from the infected mice at 35 days postinfection (dpi) were fixed in 4% paraformaldehyde at room temperature for 4 h, washed with PBSTX (1 × PBS, 0.3% Triton X-100), and treated with a permeabilization solution (50 mM DTT, 0.1% SDS, 1% NP-40 in 1 × PBS) at 37°C for 10 min. Samples were dehydrated in 100% PBSTX and graded methanol (25%, 50%, 75%, 100%), bleached in 6% bleach solution (30% H_2_O_2_ diluted in 100% methanol) under direct light at 4°C for 20 h, and rehydrated in reverse methanol gradients. Rehydrated samples were treated with proteinase K solution (1 μg/ml proteinase K, 0.1% SDS in 1 × PBS) at 37°C for 20 min, re-fixed in 4% paraformaldehyde for 10 min, and incubated with autofluorescence quenchers (Servicebio, China) for 30 min. A CY3-labeled oligonucleotide probe (S4 Table) synthesized by Huzhou Hippo Biotechnology Co., Ltd was hybridized at 56°C for 4 h. Nuclei were counterstained with DAPI, and samples were mounted with anti-fade medium. Visualization was performed on a Leica TCS SP8 Laser Scanning Confocal Microscope (Leica, Germany) using 10 × objective.

### Synthesis of dsRNA

The dsRNA templates for *SjHAT1*-dsRNA and *GFP*-dsRNA were amplified from the pGEX-4T-1-*Sj*HAT1 maintained in our lab and commercial pET-28a-GFP synthesized by Sangon Biotech (Shanghai, China), respectively, using T7 promoter-containing primers (S4 Table). The size of amplification products was verified by 1% agarose gel electrophoresis, and the identity was confirmed through DNA sequencing. Purified PCR products were employed to synthesis single-stranded RNA using the MEGAshortscript T7 Kit (Invitrogen, USA), following the manufacturer’s protocols. To synthesize dsRNA, a transcription reaction mixture containing equimolar sense and antisense ssRNA strands was denatured initially in a 75 °C water bath for 5 min, followed by annealing at room temperature for 30 min. The resulting dsRNA was subsequently treated with TURBO DNase (Invitrogen, USA) and RNase If (New England Biolabs, USA) to remove residual DNA templates and single-stranded RNA contaminants. After phenol/chloroform extraction and ethanol precipitation, the dsRNA was resuspended in DEPC-treated water. The concentration of dsRNA was quantified using a NanoDrop 2000c Spectrophotometer (Thermo fisher scientific, USA) and adjusted to 3.0 μg/μL. The integrity and size of dsRNA were verified by electrophoresis on 1% agarose gels, and the products were stored at -80 °C.

### *SjHAT1*-RNAi *in vitro*

*S. japonicum* paired adults (35 dpi) were obtained and cultured in m-AB169 (1640). *Green ﬂuorescent protein* (*GFP*) dsRNA served as the negative control for all RNAi experiments. D1 represents the first day of the experiment. Paired adults were treated with 30 μg/mL dsRNA of *GFP* or *SjHAT1* on D1, D3 and D5 with fresh medium. After daily viability assessments from D1 to D7, worms were collected for stereomicroscopy analysis (Nikon, JPN), and the viability score was assigned as described previously [51], based on the changes in mobility and general appearance. Briefly, each worm was assigned a viability score ranging from 0 to 3. Score 3: worms exhibited the highest activity level as observed in the control group, moving actively and smoothly with transparent bodies; Score 2: worms showed whole-body movement but appeared stiff and slow, with translucent bodies; Score 1: partial movement was observed with opaque body appearance; Score 0: worms remained contracted without resuming movement, considered dead. Concurrently, eggs in the culture medium were collected and enumerated on D3, D5, and D7.

### *SjHAT1*-RNAi *in vivo*

Nine *S. japonicum* infected mice were randomly divided into three groups, *SjHAT1*-dsRNA group, *GFP*-dsRNA control group, and blank control group, with three mice per group. Ten micrograms of *SjHAT1* or *GFP* dsRNA were injected into the tail vein for interference at 14 dpi, 18 dpi, 22 dpi, 26 dpi, and 30 dpi. An equal volume of 0.7% saline was injected into each mouse in the blank control group at the same time point. Following euthanasia at 35 dpi, adult worms were collected for both RNAi efficacy assessment and phenotypic analysis, while liver tissues were analyzed for egg quantification (see the section “Liver egg counting”) and histopathology (see the section “Histopathological assessment”). The experiments were repeated thrice.

### Efficacy of *SjHAT1*-RNAi

After treatment with *SjHAT1* dsRNA, transcriptional levels of *SjHAT1* of adult worms were quantified by qRT-PCR performed as previously described. Protein expression levels of *Sj*HAT1 were assessed by Western blotting. To prepare the adult worm soluble protein extracts, phosphate buffer saline precooled on ice containing 20 adult worms was homogenized by sonication (30% power, 5s on, 5s off, 2 min, Q700 Sonicator, USA), and centrifuged for 10min at 13,000 g, 4°C. Protein concentrations were determined by BCA assay. Worm extracts (20 μg protein) were separated on 10% SDS-PAGE gels and transferred to 0.45 μm PVDF membranes (Millipore, Germany). The membranes were blocked with rapid blocking buffer for 15 min at room temperature, then washed with TBST and incubated overnight with rabbit anti-*Sj*HAT1 polyclonal antibody used at 1:500 dilution in TBST at 4°C. After washing with TBST, membranes were incubated for 1 h at room temperature in TBST containing HRP-conjugated goat anti-rabbit IgG (CST, USA) at a dilution of 1:2000. After washing in TBST, the membrane was developed with BeyoECL plus solution (Beyotime Biotech, Shanghai, China) and imaged using Tanon 5200 (Tanon, Shanghai, China).

### Morphological observations of schistosomes after interference of *SjHAT1*

Adult male and female worms were fixed in 4% paraformaldehyde at room temperature overnight, gently compressed between glass slides for 1 h. Specimens were stained with hydrochloric carmine for 2 h, destained in 70% ethanol containing 1% HCl for 20 min, and dehydrated through an ethanol series (80%, 90%, 95%, 100%). After equilibration in ethanol-xylene (1:1) and clearing in 100% xylene, worms were mounted with neutral balsam on glass slides for morphometric and morphological analyses. The morphologies of reproductive organs and germ cells of *S. japonicum* were observed using a Leica TCS SP8 laser scanning confocal microscope (Leica, Germany) with a 63 × oil immersion objective.

### Scanning electron microscopy (SEM)

For SEM analysis, worms were fixed in 1% osmium tetroxide and dehydrated in graded ethanol. After that, the specimens were dried for approximately 30 min. Then the worm samples were mounted on aluminum stubs, coated with gold, and examined with a Hitachi-S4700 scanning electron microscope (Chiyodaku, Japan).

### Liver egg counting

0.5g of mouse liver was weighed and incubated overnight in 5% sodium hydroxide (NaOH) at 37°C and 220 rpm to obtain a homogeneous solution. Ten μL of the above solution was observed and counted under an ordinary upright microscope. Each sample was counted 10 times, and the average was calculated. Each group had at least three replicates. The number of liver eggs (EPG) was calculated using the formula: EPG = average×1000/ 0.5

### Histopathological assessment

The liver of each mouse was washed with PBS (pH 7.4) and fixed with paraformaldehyde. After dehydration, the tissues were embedded in paraffin. Sections 4-μm-thick were stained with hematoxylin and eosin for granuloma analysis. The areas of granulomas surrounding single egg were observed at 200 × magnification.

### Statistics

Data were analyzed with GraphPad Prism or Excel, expressed as means ± SD, and tested for statistical significance using either ANOVA or *t*-tests, as noted in the figure legends.

### RNA sequencing

Worms incubated with *SjHAT1*- and *GFP*-dsRNA for 7 days were collected and washed three times with 1 × PBS (pH 7.4). After quick freezing in liquid nitrogen, the samples were sent to SeqHealth Technology Corporation (Wuhan, China) for RNA extraction, library construction and sequencing. RNA integrity was validated using the LabChip GX Touch system (PerkinElmer, USA), followed by fluorometric quantification via Qubit 3.0 Fluorometer (Thermo Fisher Scientific, USA). Libraries were constructed from 500 ng total RNA using the KC mRNA Library Prep Kit (Seqhealth, China) according to the manufacturer’s protocol and sequencing was performed on the DNBSEQ-T7 platform (MGI, China).

### Analysis of gene expression profiles and differential expression genes

The FASTQC program (https://www.bioinformatics.babraham.ac.uk/projects/fastqc/) performed quality control on Raw data. FASTP v0.23.2 was used to remove low-quality reads and trim adapter sequences to generate clean reads. Clean reads were aligned to the *S. japonicum* genome (ASM2146165v1) using STAR v2.7.6a, followed by exonic read quantification with featureCounts (Subread-1.5.1) and RPKM normalization for gene expression profiling. Differential expression analysis was performed using the edgeR package (v3.40.2) with thresholds of fold change (FC)>1.5 or ＜1.5 and *p* < 0.05. Statistically significant GO terms and KEGG pathways enriched in differentially expressed genes (DEGs) were identified using KOBAS v2.1.1 with a significance threshold of *p* < 0.05. Transcriptional validation of target genes was performed through qRT-PCR as previously described, with primer sequences listed in S4 Table.

## Supporting information

S1 Fig Structural prediction and validation of *Sj*HAT1. (A) Predicted three-dimensional structure of *Sj*HAT1 color-coded by predicted local distance difference test (pLDDT) values, along with the predicted aligned error (PAE) matrix. Most amino acid residues exhibits pLDDT scores exceeding 80, indicating high reliability at the local structural level. The predicted TM-score (pTM) of 0.86 suggests accurate global folding of the protein. The PAE matrix shows consistently low error values, reflecting stable spatial arrangements between structural domains. (B) Ramachandran plot showing the distribution of backbone dihedral angles. Approximately 91.0% of residues are located in the favored regions of the Ramachandran plot, with none in disallowed regions, indicating excellent stereochemical quality of the protein backbone.
(DOCX)

S2 Fig Efficiency of RNA interference for *SjHAT1*-dsRNA. Adult female and male worms with good activity were added to the 24-well plate with five worm per well, different kinds of *SjHAT1*-dsRNA were added for interference on the ﬁrst, third, and ﬁfth days. Each group had three replicate wells for each biological experiment, with three biological replicates. DEPC-treated water served as the blank control, and *green ﬂuorescent protein* (*GFP*) dsRNA was used as the negative control. (**A**) Comparison of RNA interference efficiency among *SjHAT1*-dsRNA1 (30 μg/mL), *SjHAT1*-dsRNA2 (30 μg/mL), and *SjHAT1*-dsRNA1/2 (15 μg/mL *SjHAT1*-dsRNA1 + 15 μg/mL *SjHAT1*-dsRNA2) in female worms. (**B**) Comparison of RNA interference efficiency among *SjHAT1*-dsRNA1, *SjHAT1*-dsRNA2, and *SjHAT1*-dsRNA1/2 in male worms. Error bars indicate standard deviation (SD), n = 3 biological replicates, One-Way ANOVA, ‘ns’, not significant, *p < 0.05, **p < 0.01. The raw data is shown in supporting information file [Table I in S1 Data].
(DOCX)

S3 Fig Effect of *SjHAT1*-dsRNA on worm pairing. Adults with good pairing activity were incubated in the 24-well plate with three pairs per well. Each group had two replicate wells for each biological experiment, with four biological replicates. **(A**) Effect of *SjHAT1*-dsRNA on pairing rate. The raw data is shown in supporting information file [Table J in S1 Data]. (**B**) Observation under the light microscope on D7 after treatment with *GFP*-dsRNA and *SjHAT1*-dsRNA. Error bars indicate standard deviation (SD). *p < 0.01 (Two-Way ANOVA).
(DOCX)

S4 Fig Scanning electron micrographs of the tegument of *S. japonicum* females in the control group after 3 days incubation. (A) Gentle panorama of a female worm. (B) Anterior part of the female worm. (C) Mid-portion of the female worm. (D) Spines in the ventral sucker of the female. (E)-(F) Transverse ridges on the tegument of the female worm. Scalebars: A: 1 mm; B: 100 μm; C: 100 μm; D: 5 μm; E: 10 μm; F: 3 μm.
(DOCX)

S5 Fig Scanning electron micrographs of the tegument of female *S. japonicum* worms after treatment with 100 μM DW-3–15 for 72 h. (A)The whole worm is swollen and shrunken; (B) extensive sloughing is observed on the tegument in the mid-body of female worm; (C) severe swelling accompanied by numerous blisters is observed at the anterior end of female worm; (D) fusion of the spines in the ventral sucker are obvious; (E)-(F) the ridge-like structures on the tegument of female worm are swollen. Scalebars: A: 1 mm; B: 100 μm; C: 100 μm; D: 5 μm; E: 10 μm; F: 3 μm.
(DOCX)

S6 Fig *GFP* RNAi has no effect on worm survival, worm pairing and oviposition *in vivo.* On the 14th, 18th, 22th, 26th and 30th days postinfection, *GFP*-dsRNA was injected through the tail vein, and the hepatic portal vein was perfused on the 35^th^ day. (A) Worm burden of the parasites recovered at 35 dpi in Blank and *GFP* RNAi groups. N = 3 biological replicates, 3 mice of each biological replicates. Student’s *t*-test, ‘ns’, not significant. The raw data is shown in supporting information file [Table K in S1 Data]. (B) Comparation of the pairing rates of recovered worms between blank control group and *GFP* RNAi group. N = 3 biological replicates, 3 mice of each biological replicates. Student’s *t*-test, ‘ns’, not significant. The raw data is shown in supporting information file [Table L in S1 Data]. (C) Observation of the reproductive organs of worms after *in vivo* treatment. ‘t’, testis; ‘ov’, ovary; ‘vg’, vitelline gland. Scale bars: 20 μm. (D) Gross observations of the mouse liver from the blank and *GFP* RNAi group. Scale bars: 1 cm. (E) Egg count per gram of liver comparation between the blank and *GFP* RNAi groups (n = 90), Student’s *t*-test, ‘ns’, not significant. The raw data is shown in supporting information file [Table M in S1 Data]. (F) Histological assessment of mouse liver by H&E staining. Scale bars: 100 μm. (G) Statistical analysis of the size of egg granuloma area after *in vivo* treatment (n = 30), Student’s *t*-test, ‘ns’, not significant. Error bars indicate standard deviation (SD). The raw data is shown in supporting information file [Table N in S1 Data].
(DOCX)

S1 Table Effect of DW-3–15 on the enzymatic activity of recombinant *Hs*HAT1. *r*Hs*HAT1 activity (pmol/min)=ΔAFU/[time(min)*the slope of the standard curve]. ΔAFU = AFU(sample)-AFU(background). The regression equation of the Standard Curve is Y = 9.7537X + 131.71, R^2^ = 0.9967. Histone Acetyltransferase Assay Kit (Fluorescent) and r*Hs*HAT1 were purchased from Active Motif (Shanghai, China). The raw data is shown in supporting information file [Table O in S1 Data].
(DOCX)

S2 Table Structural validation statistics of the predicted protein model. *MolProbity analysis reveals a MolProbity score of 1.52 and a clashscore of 4.38, both characteristic of high-quality structures. Side-chain conformation analysis demonstrates that 98.74% of residues adopt favored rotamer conformations, whereas only 0.25% categorized as poor rotamers. ERRAT analysis yields an overall quality factor of 95.78, indicative of reliable non-bonding atomic interactions. Verify3D analysis confirms that 78.98% of residues have good compatibility between the three-dimensional structure and the amino acid sequence.
(DOCX)

S3 Table The number of mature and immature female worms recovered on 35^th^ day post RNA interference treatment with *GFP*-dsRNA and *SjHAT1*-dsRNA. Chi-square test: *χ*^2^(1) =5.528, *p* = 0.0187.
(DOCX)

S4 Table Primers list. (DOCX)

S1 Data Raw data for the Figs and Tables. (RAR)
