# Supplementary material for: Schistosoma japonicum histone acetyltransferase 1 (SjHAT1): A novel anti-schistosomal drug target
Source: PLoS Pathog. 2026 Jun 24;22(6):e1014334. doi: 10.1371/journal.ppat.1014334 (PMC13293438; doi:10.1371/journal.ppat.1014334)
Supplement: S1 Fig — (A) Predicted three-dimensional structure of SjHAT1 color-coded by predicted local distance difference test (pLDDT) values, along with the predicted aligned error (PAE) matrix. Most amino acid residues exhibits pLDDT scores exceeding 80, indicating high reliability at the local structural level. The predicted TM-score (pTM) of 0.86 suggests accurate global folding of the protein. The PAE matrix shows consistently low error values, reflecting stable spatial arrangements between structural domains. (B) Ramachandran plot showing the distribution of backbone dihedral angles. Approximately 91.0% of residues are located in the favored regions of the Ramachandran plot, with none in disallowed regions, indicating excellent stereochemical quality of the protein backbone. (DOCX) [file ppat.1014334.s001.docx]

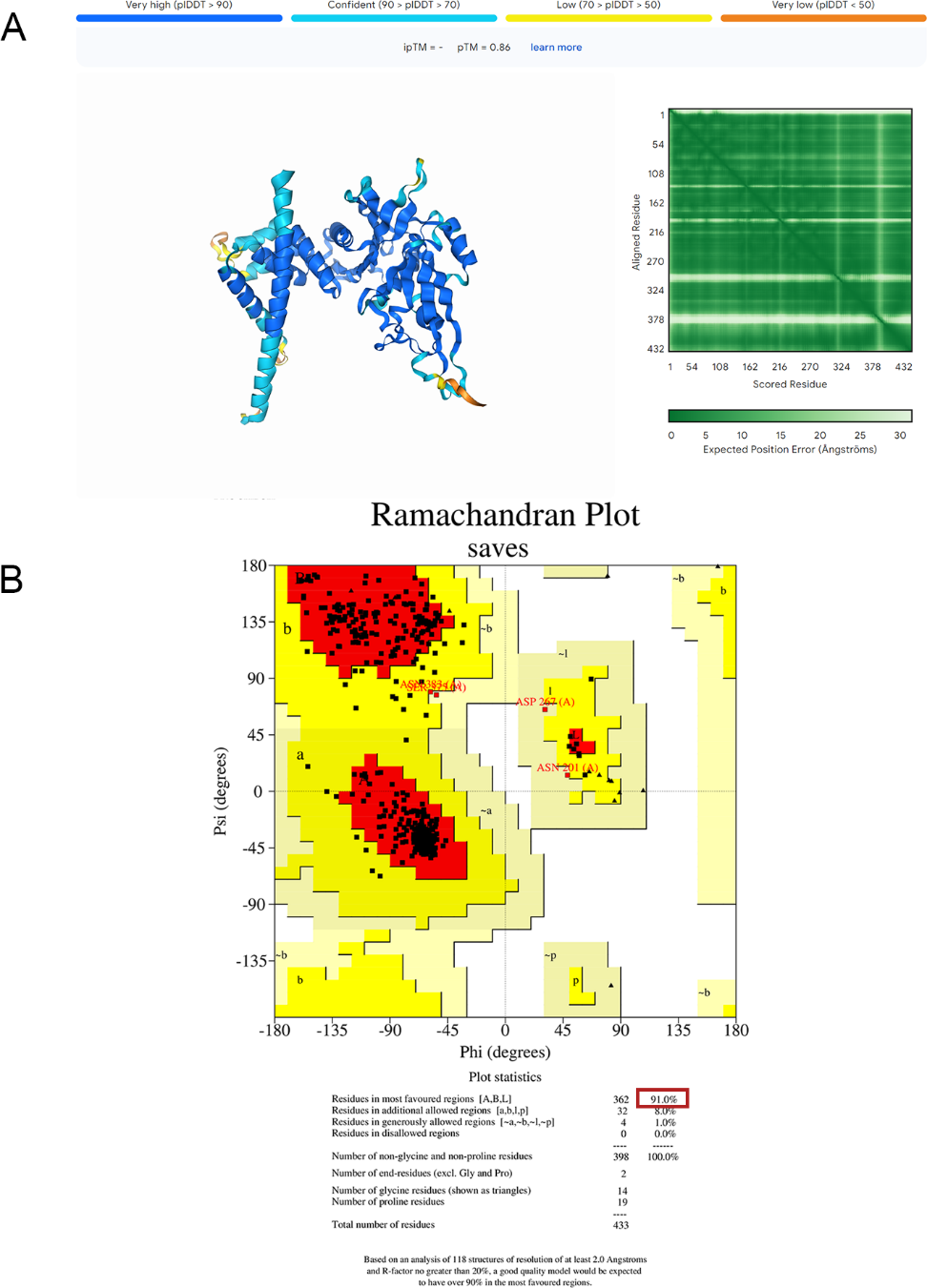


**S1 Fig. Structural prediction and validation of SjHAT1.** (A) Predicted three-dimensional structure of SjHAT1 color-coded by predicted local distance difference test (pLDDT) values, along with the predicted aligned error (PAE) matrix. Most amino acid residues exhibits pLDDT scores exceeding 80, indicating high reliability at the local structural level. The predicted TM-score (pTM) of 0.86 suggests accurate global folding of the protein. The PAE matrix shows consistently low error values, reflecting stable spatial arrangements between structural domains. (B) Ramachandran plot showing the distribution of backbone dihedral angles. Approximately 91.0% of residues are located in the favored regions of the Ramachandran plot, with none in disallowed regions, indicating excellent stereochemical quality of the protein backbone.
