# Supplementary material for: Schistosoma japonicum histone acetyltransferase 1 (SjHAT1): A novel anti-schistosomal drug target
Source: PLoS Pathog. 2026 Jun 24;22(6):e1014334. doi: 10.1371/journal.ppat.1014334 (PMC13293438; doi:10.1371/journal.ppat.1014334)
Supplement: S2 Fig — Adult female and male worms with good activity were added to the 24-well plate with five worm per well, different kinds of SjHAT1-dsRNA were added for interference on the ﬁrst, third, and ﬁfth days. Each group had three replicate wells for each biological experiment, with three biological replicates. DEPC-treated water served as the blank control, and green ﬂuorescent protein (GFP) dsRNA was used as the negative control. (A) Comparison of RNA interference efficiency among SjHAT1-dsRNA1 (30 μg/mL), SjHAT1-dsRNA2 (30 μg/mL), and SjHAT1-dsRNA1/2 (15 μg/mL SjHAT1-dsRNA1 + 15 μg/mL SjHAT1-dsRNA2) in female worms. (B) Comparison of RNA interference efficiency among SjHAT1-dsRNA1, SjHAT1-dsRNA2, and SjHAT1-dsRNA1/2 in male worms. Error bars indicate standard deviation (SD), n = 3 biological replicates, One-Way ANOVA, ‘ns’, not significant, *p < 0.05, **p < 0.01. The raw data is shown in supporting information file [Table I in S1 Data]. (DOCX) [file ppat.1014334.s002.docx]

**S2 Fig. Efficiency of RNA interference for** ***SjHAT1*-dsRNA.** Adult female and male worms with good activity were added to the 24-well plate with five worm per well, different kinds of *SjHAT1*-dsRNA were added for interference on the ﬁrst, third, and ﬁfth days. Each group had three replicate wells for each biological experiment, with three biological replicates. DEPC-treated water served as the blank control, and *green ﬂuorescent protein* (*GFP*) dsRNA was used as the negative control. (**A**) Comparison of RNA interference efficiency among *SjHAT1*-dsRNA1 (30 μg/mL), *SjHAT1*-dsRNA2 (30 μg/mL), and *SjHAT1*-dsRNA1/2 (15 μg/mL *SjHAT1*-dsRNA1+15 μg/mL *SjHAT1*-dsRNA2) in female worms. (**B**) Comparison of RNA interference efficiency among *SjHAT1*-dsRNA1, *SjHAT1*-dsRNA2, and *SjHAT1*-dsRNA1/2 in male worms. Error bars indicate standard deviation (SD), n = 3 biological replicates, One-Way ANOVA, ‘ns’, not significant, **p* < 0.05, ***p* <0.01. The raw data is shown in supporting information file [S1 Data] named as raw data for S2 Fig.
