# Supplementary material for: Schistosoma japonicum histone acetyltransferase 1 (SjHAT1): A novel anti-schistosomal drug target
Source: PLoS Pathog. 2026 Jun 24;22(6):e1014334. doi: 10.1371/journal.ppat.1014334 (PMC13293438; doi:10.1371/journal.ppat.1014334)
Supplement: S3 Fig — Adults with good pairing activity were incubated in the 24-well plate with three pairs per well. Each group had two replicate wells for each biological experiment, with four biological replicates. (A) Effect of SjHAT1-dsRNA on pairing rate. The raw data is shown in supporting information file [Table J in S1 Data]. (B) Observation under the light microscope on D7 after treatment with GFP-dsRNA and SjHAT1-dsRNA. Error bars indicate standard deviation (SD). *p < 0.01 (Two-Way ANOVA). (DOCX) [file ppat.1014334.s003.docx]

**S3 Fig. Effect of *SjHAT1*-dsRNA on worm pairing.** Adults with good pairing activity were incubated in the 24-well plate with three pairs per well. Each group had two replicate wells for each biological experiment, with four biological replicates. **(A**) Effect of *SjHAT1*-dsRNA on pairing rate. The raw data is shown in supporting information file [S1 Data] named as raw data for S3A Fig. (**B**) Observation under the light microscope on D7 after treatment with *GFP*-dsRNA and *SjHAT1*-dsRNA. Error bars indicate standard deviation (SD). **p* < 0.01 (Two-Way ANOVA).
