# Supplementary material for: Schistosoma japonicum histone acetyltransferase 1 (SjHAT1): A novel anti-schistosomal drug target
Source: PLoS Pathog. 2026 Jun 24;22(6):e1014334. doi: 10.1371/journal.ppat.1014334 (PMC13293438; doi:10.1371/journal.ppat.1014334)
Supplement: S4 Fig — (A) Gentle panorama of a female worm. (B) Anterior part of the female worm. (C) Mid-portion of the female worm. (D) Spines in the ventral sucker of the female. (E)-(F) Transverse ridges on the tegument of the female worm. Scalebars: A: 1 mm; B: 100 μm; C: 100 μm; D: 5 μm; E: 10 μm; F: 3 μm. (DOCX) [file ppat.1014334.s004.docx]

**S4 Fig. Scanning electron micrographs of the tegument of *S. japonicum* females in the control group after 3 days incubation.** (A) Gentle panorama of a female worm. (B) Anterior part of the female worm. (C) Mid-portion of the female worm. (D) Spines in the ventral sucker of the female. (E)-(F) Transverse ridges on the tegument of the female worm. Scalebars: A: 1 mm; B: 100 μm; C: 100 μm; D: 5 μm; E: 10 μm; F: 3 μm.
