# Supplementary material for: Schistosoma japonicum histone acetyltransferase 1 (SjHAT1): A novel anti-schistosomal drug target
Source: PLoS Pathog. 2026 Jun 24;22(6):e1014334. doi: 10.1371/journal.ppat.1014334 (PMC13293438; doi:10.1371/journal.ppat.1014334)
Supplement: S5 Fig — (A)The whole worm is swollen and shrunken; (B) extensive sloughing is observed on the tegument in the mid-body of female worm; (C) severe swelling accompanied by numerous blisters is observed at the anterior end of female worm; (D) fusion of the spines in the ventral sucker are obvious; (E)-(F) the ridge-like structures on the tegument of female worm are swollen. Scalebars: A: 1 mm; B: 100 μm; C: 100 μm; D: 5 μm; E: 10 μm; F: 3 μm. (DOCX) [file ppat.1014334.s005.docx]

**S5 Fig. Scanning electron micrographs of the tegument of female S. japonicum worms after treatment with 100 μM DW-3-15 for 72 h.** (A)The whole worm is swollen and shrunken; (B) extensive sloughing is observed on the tegument in the mid-body of female worm; (C) severe swelling accompanied by numerous blisters is observed at the anterior end of female worm; (D) fusion of the spines in the ventral sucker are obvious; (E)-(F) the ridge-like structures on the tegument of female worm are swollen. Scalebars: A: 1 mm; B: 100 μm; C: 100 μm; D: 5 μm; E: 10 μm; F: 3 μm.
