# Supplementary material for: Schistosoma japonicum histone acetyltransferase 1 (SjHAT1): A novel anti-schistosomal drug target
Source: PLoS Pathog. 2026 Jun 24;22(6):e1014334. doi: 10.1371/journal.ppat.1014334 (PMC13293438; doi:10.1371/journal.ppat.1014334)
Supplement: S6 Fig — On the 14th, 18th, 22th, 26th and 30th days postinfection, GFP-dsRNA was injected through the tail vein, and the hepatic portal vein was perfused on the 35th day. (A) Worm burden of the parasites recovered at 35 dpi in Blank and GFP RNAi groups. N = 3 biological replicates, 3 mice of each biological replicates. Student’s t-test, ‘ns’, not significant. The raw data is shown in supporting information file [Table K in S1 Data]. (B) Comparation of the pairing rates of recovered worms between blank control group and GFP RNAi group. N = 3 biological replicates, 3 mice of each biological replicates. Student’s t-test, ‘ns’, not significant. The raw data is shown in supporting information file [Table L in S1 Data]. (C) Observation of the reproductive organs of worms after in vivo treatment. ‘t’, testis; ‘ov’, ovary; ‘vg’, vitelline gland. Scale bars: 20 μm. (D) Gross observations of the mouse liver from the blank and GFP RNAi group. Scale bars: 1 cm. (E) Egg count per gram of liver comparation between the blank and GFP RNAi groups (n = 90), Student’s t-test, ‘ns’, not significant. The raw data is shown in supporting information file [Table M in S1 Data]. (F) Histological assessment of mouse liver by H&E staining. Scale bars: 100 μm. (G) Statistical analysis of the size of egg granuloma area after in vivo treatment (n = 30), Student’s t-test, ‘ns’, not significant. Error bars indicate standard deviation (SD). The raw data is shown in supporting information file [Table N in S1 Data]. (DOCX) [file ppat.1014334.s006.docx]

**S6 Fig. *GFP* RNAi has no effect on worm survival, worm pairing and oviposition *in vivo****.* On the 14th, 18th, 22th, 26th and 30th days postinfection, GFP-dsRNA was injected through the tail vein, and the hepatic portal vein was perfused on the 35th day. (A) Worm burden of the parasites recovered at 35 dpi in Blank and GFP RNAi groups. N = 3 biological replicates, 3 mice of each biological replicates. Student’s t-test, ‘ns’, not significant. The raw data is shown in supporting information file [S1 Data] named as raw data for S6A Fig. (B) Comparation of the pairing rates of recovered worms between blank control group and GFP RNAi group. N = 3 biological replicates, 3 mice of each biological replicates. Student’s t-test, ‘ns’, not significant. The raw data is shown in supporting information file [S1 Data] named as raw data for S6B Fig. (C) Observation of the reproductive organs of worms after in vivo treatment. ‘t’, testis; ‘ov’, ovary; ‘vg’, vitelline gland. Scale bars: 20 μm. (D) Gross observations of the mouse liver from the blank and GFP RNAi group. Scale bars: 1 cm. (E) Egg count per gram of liver comparation between the blank and GFP RNAi groups (n = 90), Student’s t-test, ‘ns’, not significant. The raw data is shown in supporting information file [S1 Data] named as raw data for S6E Fig. (F) Histological assessment of mouse liver by H&E staining. Scale bars: 100 μm. (G) Statistical analysis of the size of egg granuloma area after in vivo treatment (n = 30), Student’s t-test, ‘ns’, not significant. Error bars indicate standard deviation (SD). The raw data is shown in supporting information file [S1 Data] named as raw data for S6G Fig.
