# Supplementary material for: Schistosoma japonicum histone acetyltransferase 1 (SjHAT1): A novel anti-schistosomal drug target
Source: PLoS Pathog. 2026 Jun 24;22(6):e1014334. doi: 10.1371/journal.ppat.1014334 (PMC13293438; doi:10.1371/journal.ppat.1014334)
Supplement: S1 Table — *rHsHAT1 activity (pmol/min)=ΔAFU/[time(min)*the slope of the standard curve]. ΔAFU = AFU(sample)-AFU(background). The regression equation of the Standard Curve is Y = 9.7537X + 131.71, R2 = 0.9967. Histone Acetyltransferase Assay Kit (Fluorescent) and rHsHAT1 were purchased from Active Motif (Shanghai, China). The raw data is shown in supporting information file [Table O in S1 Data]. (DOCX) [file ppat.1014334.s007.docx]

**S1 Table. Effect of DW-3-15 on** **the enzymatic activity of recombinant *Hs*HAT1**

| Group | ΔAFU | ΔT (min) | CoA production(pmol) | Acetyltransferase (HAT) activity (μU/mL)^*^ |
| --- | --- | --- | --- | --- |
| Background | 0 | 30 | 0 | 0 |
| Positive control | 3006.3 | 30 | 294.7 | 10.3 |
| 0.125μg rHAT1 | 10275.6 | 30 | 1040.0 | 35.1 |
| 0.125μg r*Hs*HAT1+0.5 μM DW-3-15 | 10054.2 | 30 | 1017.3 | 34.4 |
| 0.125μg r*Hs*HAT1+5 μM DW-3-15 | 10677.8 | 30 | 1081.2 | 36.5 |
| 0.125μg r*Hs*HAT1+50 μM DW-3-15 | 10506.3 | 30 | 1063.7 | 35.9 |

*r*Hs*HAT1 activity (pmol/min)=ΔAFU/[time(min)*the slope of the standard curve]. ΔAFU= AFU(sample)-AFU(background). The regression equation of the Standard Curve is Y = 9.7537X + 131.71, R^2^=0.9967. Histone Acetyltransferase Assay Kit (Fluorescent) and r*Hs*HAT1 were purchased from Active Motif (Shanghai, China). The raw data is shown in supporting information file [S1 Data] named as raw data for S1 Table.
