# Supplementary material for: Schistosoma japonicum histone acetyltransferase 1 (SjHAT1): A novel anti-schistosomal drug target
Source: PLoS Pathog. 2026 Jun 24;22(6):e1014334. doi: 10.1371/journal.ppat.1014334 (PMC13293438; doi:10.1371/journal.ppat.1014334)
Supplement: S2 Table — *MolProbity analysis reveals a MolProbity score of 1.52 and a clashscore of 4.38, both characteristic of high-quality structures. Side-chain conformation analysis demonstrates that 98.74% of residues adopt favored rotamer conformations, whereas only 0.25% categorized as poor rotamers. ERRAT analysis yields an overall quality factor of 95.78, indicative of reliable non-bonding atomic interactions. Verify3D analysis confirms that 78.98% of residues have good compatibility between the three-dimensional structure and the amino acid sequence. (DOCX) [file ppat.1014334.s008.docx]

**S2 Table. Structural validation statistics of the predicted protein model**

| Validation method | Parameter | Value^*^ |
| --- | --- | --- |
| MolProbity | MolProbity score | 1.52 |
| MolProbity | Clashscore | 4.38 |
| MolProbity | Favored rotamers (%) | 98.74 |
| MolProbity | Poor rotamers (%) | 0.25 |
| MolProbity | Cβ deviations (>0.25 Å) | 0 |
| MolProbity | Bad bonds (%) | 0.00 |
| MolProbity | Bad angles (%) | 0.41 |
| ERRAT | Overall quality factor | 95.78 |
| Verify3D | Residues ≥0.1 (%) | 78.98 |

*****MolProbity analysis reveals a MolProbity score of 1.52 and a clashscore of 4.38, both characteristic of high-quality structures. Side-chain conformation analysis demonstrates that 98.74% of residues adopt favored rotamer conformations, whereas only 0.25% categorized as poor rotamers. ERRAT analysis yields an overall quality factor of 95.78, indicative of reliable non-bonding atomic interactions. Verify3D analysis confirms that 78.98% of residues have good compatibility between the three-dimensional structure and the amino acid sequence.
