# Supplementary material for: Schistosoma japonicum histone acetyltransferase 1 (SjHAT1): A novel anti-schistosomal drug target
Source: PLoS Pathog. 2026 Jun 24;22(6):e1014334. doi: 10.1371/journal.ppat.1014334 (PMC13293438; doi:10.1371/journal.ppat.1014334)
Supplement: S3 Table — Chi-square test: χ2(1) =5.528, p = 0.0187. (DOCX) [file ppat.1014334.s009.docx]

**S3 Table. The number of mature and immature female worms recovered on 35^th^ day post RNA interference treatment with *GFP*-dsRNA and *SjHAT1*-dsRNA**

| group | Number of mature female‌ worm | Number of immature female worm |
| --- | --- | --- |
| *GFP* RNAi | 138 | 32 |
| *SjHAT1* RNAi | 47 | 23 |

Chi-square test: *χ*^2^(1) =5.528, *p*=0.0187
