# Supplementary material for: Schistosoma japonicum histone acetyltransferase 1 (SjHAT1): A novel anti-schistosomal drug target
Source: PLoS Pathog. 2026 Jun 24;22(6):e1014334. doi: 10.1371/journal.ppat.1014334 (PMC13293438; doi:10.1371/journal.ppat.1014334)
Supplement: S4 Table — (DOCX) [file ppat.1014334.s010.docx]

**S4 Table. Primers list**

| RACE | |
| --- | --- |
| RACE primer (5’-3’) | Primer sequences |
| 3’GSP | ATCCAAAGGTGTTGCGCCTGATGATATT |
| 3’NGSP | GATTACGCCAAGCTTAGACCCATCACCTGATTTCCAACG |
| 5’GSP | AGTTTCCAGGCAACGTTTGCAGTCCAGT |
| 5’NGSP | GATTACGCCAAGCTTGCAGTCCAGTACATCTCTAATACGTTGG |
|  |  |
| Quantitative of *SjHAT1* transcript levels | |
| Primer name (5’-3’) | Primer sequence |
| *PSMD4*-qF | CCTCACCAACAATTTCCACATCT |
| *PSMD4*-qR | GATCACTTATAGCCTTGCGAACAT |
| *SjHAT1*-qF | GCAGATGACATTCAGTGGTGCTATT |
| *SjHAT1*-qR | AGGTGGAAGGATAAGAACTTGGCT |
|  |  |
| FISH | |
| Probe name (5’-3’) | Probe sequence |
| *SjHAT1* probe | CY3-TCTAAAAGGTGGAAGGATAAGAACTTGGCTAACA |
|  |  |
| Synthesis of dsRNA | |
| Primer name (5’-3’) | Primer sequence |
| *GFP*-dsRNA-F | TAATACGACTCACTATAGGGAGAAAGCAGAAGAACGGCATCAAG |
| *GFP*-dsRNA-R | TAATACGACTCACTATAGGGAGACGAACTCCAGCAGGACCAT |
| *SjHAT1*-dsRNA1-F | TAATACGACTCACTATAGGGAGACGGCCCTCAACTATCCA |
| *SjHAT1*-dsRNA1-R | TAATACGACTCACTATAGGGAGACAGGCATACCATGTTCG |
| *SjHAT1*-dsRNA2-F | TAATACGACTCACTATAGGGAGAGACTGCAAACGTTGCCTG |
| *SjHAT1*-dsRNA2-R | TAATACGACTCACTATAGGGAGAGAAAGTACGCAGTAGCGC |
|  |  |
| Quantitative of of *AADC* and *MRP4* transcript levels | |
| Primer name (5’-3’) | Primer sequence |
| *AADC*-qF | ACAATGGGTTTACGTGATCGAATGC |
| *AADC*-qR | CCGAAGTACCGAACTATGTGCTTGA |
| *MRP4*-qF | GGCTCTGGATTAAGTGGTGGTCAA |
| *MRP4*-qR | CCGCCGCTAAAGGGTCATCTAAA |
