# Supplementary material for: Schistosoma japonicum histone acetyltransferase 1 (SjHAT1): A novel anti-schistosomal drug target
Source: PLoS Pathog. 2026 Jun 24;22(6):e1014334. doi: 10.1371/journal.ppat.1014334 (PMC13293438; doi:10.1371/journal.ppat.1014334)
Supplement: Attachment — Submitted filename: Response to reviewers.docx [file ppat.1014334.s012.docx]

Dear Editors,

Thanks very much for taking your time to review this manuscript. We thank the editors and reviewers for the time and effort that they have put into reviewing the previous version of the manuscript. Their suggestions have enabled us to improve our work. Based on the instructions provided in your letter, we uploaded the clean file of the revised manuscript. Accordingly, we have uploaded a copy of the original manuscript with all the changes highlighted by using the track changes mode.

Appended to this letter is our point-by-point response to the comments raised by the reviewers. The comments are reproduced and our responses are given directly afterward in a different color (blue).

We would like also to thank you for allowing us to resubmit a revised copy of the manuscript.

We hope that the revised manuscript will meet the approval for publication in the Journal of ***PLOS Pathogens***.

Sincerely,

Chao-Ming Xia

Department of Parasitology

Suzhou Medical College of Soochow University

Industrial Park, Suzhou, Jiangsu province 215123, P. R. China

E-mail: xiachaoming@suda.edu.cn

Phone: +86 13861325729; fax: +86 0512 65880103

**Suggestions from the reviewer:**

Point 1: The authors have the complete coding sequence for *Sj*HAT1. They present much useful and interesting data on the effects seen in worms resulting from *Sj*HAT1 knock downs, but no evidence is presented directly linking DW-3-15 to inhibition of *Sj*HAT1. To establish this mechanism of action, recombinant *Sj*HAT1 should be produced and direct inhibition of its enzymatic activity by DW-3-15 should be shown. Short of this, the results presented are too speculative.

Answer: We thank the reviewer for this constructive suggestion. It is true that lack of evidence linking DW-3-15 to inhibition of *Sj*HAT1 may compromise the persuasiveness of our results. To address this, we successfully expressed recombinant *Sj*HAT1 (r*Sj*HAT1) in *E. coli* (Line 518-537), and demonstrated that recombinant protein exhibited robust histone acetyltransferase activity (Line 538-552). Furthermore, we showed that DW-3-15 dose-dependently inhibited the enzymatic activity of r*Sj*HAT1, with complete inhibition observed at 50 μM (Line 34-37, Line 153-157, Line169-175, Table 1, Line 376-380). These results confirm *Sj*HAT1 as a novel druggable target.

**Table 1. Enzymatic activity of recombinant *Sj*HAT1**

| Group | ΔRFU | ΔT (min) | CoA^**^ production(pmol) | Acetyltransferase (HAT) activity (μU/mL)^***^ |
| --- | --- | --- | --- | --- |
| Background | 0 | 55 | 0 | 0 |
| Positive control | 235353 | 55 | 493 | 3 |
| r*Sj*HAT1^*^ (2.5μL) | 96077 | 55 | 178 | 130 |
| r*Sj*HAT1 (2.5μL) + 0.5 μM DW-3-15 | 30087 | 55 | 29 | 21 |
| r*Sj*HAT1 (2.5μL) + 5 μM DW-3-15 | 21095 | 55 | 9 | 6 |
| r*Sj*HAT1 (2.5μL) + 50 μM DW-3-15 | 11492 | 55 | 0 | 0 |

*The initial concentration of r*Sj*HAT1 was 2.03 mg/mL. After a 100-fold dilution, the addition of 2.5 μl resulted in an r*Sj*HAT1 content of 50 ng.

**The regression equation is Y = 442.58X + 17262, R^2^=0.99953.

***HAT activity (µU/mL ) = [B/(∆T×V)] × D. B = Amount of CoA from standard curve (pmol). ΔRFU was used to to obtain B pmol of CoA generated by histone acetyltransferase during the reaction time. ΔT = reaction time (min). V = Original sample volume added into the reaction well (µL). D = Sample dilution factor.

Point 2: If the target of DW-3-15 is *Sj*HAT1, then morphological changes resulting from silencing *Sj*HAT1should resemble morphological changes resulting from DW-3-15 treatment. No results showing phenotypic changes after treatment with DW-3-15 were presented. An earlier study in *S. mansoni* using HAT inhibitor PU139 was cited, but this is not sufficient to link DW-3-15 to the morphological changes seen with *Sj*HAT1 silencing.

Answer: We thank the reviewer for this insightful suggestion. We have shown the morphological changes following treatment with DW-3-15 in Supplementary S4 Fig. Our scanning electron microscope analysis reveals that *SjHAT1* silencing induces morphological alterations similar to those observed after 72 h treatment with 100 μM DW-3-15, albeit with less severe damage compared to the DW-3-15 treatment group (Line 244-247, Line 401-402, S4 Fig).

**S4 Fig. Scanning electron micrographs of the tegument of female *S. japonicum* worms after treatment with 100 μM DW-3-15 for 72 h.** (A)The whole worm is swollen and shrunken; (B) extensive sloughing is observed on the tegument in the mid-body of female worm; (C) severe swelling accompanied by numerous blisters is observed at the anterior end of female worm; (D) fusion of the spines in the ventral sucker are obvious; (E)-(F) the ridge-like structures on the tegument of female worm are swollen. Scalebars: A: 1 mm; B: 100 μm; C: 100 μm; D: 5 μm; E: 10 μm; F: 3 μm.

Our *in vitro* and *in vivo* experiments demonstrated that knockdown of *SjHAT1* not only compromised worm survival but also inhibited oviposition in female schistosome worms. The downregulation of *SjHAT1* significantly reduced egg production in female worms, potentially mediated by a dual-sex regulatory mechanism. This mechanism highlights *Sj*HAT1’s pivotal role in schistosome reproduction and survival, providing novel molecular targets for transmission-blocking interventions against schistosomiasis.
