# Supplementary material for: Schistosoma japonicum histone acetyltransferase 1 (SjHAT1): A novel anti-schistosomal drug target
Source: PLoS Pathog. 2026 Jun 24;22(6):e1014334. doi: 10.1371/journal.ppat.1014334 (PMC13293438; doi:10.1371/journal.ppat.1014334)
Supplement: Attachment — Submitted filename: Response to Reviewers-new.docx [file ppat.1014334.s013.docx]

Dear Editors,

Thanks very much for taking your time to review this manuscript. We thank the editors and reviewers for the time and effort that they have put into reviewing the previous version of the manuscript. Their suggestions have enabled us to improve our work. Based on the instructions provided in your letter, we have uploaded a marked-up copy of our manuscript that highlights changes made to the original version, labeled as ‘Revised Manuscript with Track Changes. Accordingly, an unmarked version of our revised paper without tracked changes has been uploaded as a separate file labeled ‘Manuscript’.

Appended to this letter is our point-by-point response to the comments raised by the editors and reviewers. The comments are reproduced and our responses are given directly afterward in a different color (blue).

We would like also to thank you for allowing us to resubmit a revised copy of the manuscript.

We hope that the revised manuscript will meet the approval for publication in the Journal of PLOS Pathogens.

Sincerely,

Chao-Ming Xia

Department of Parasitology

Suzhou Medical College of Soochow University

Industrial Park, Suzhou, Jiangsu province 215123, P. R. China

E-mail: xiachaoming@suda.edu.cn

Phone: +86 0512 65880133; fax: +86 0512 65880103

**Suggestions from Editors,**

The reviewers find the experimental work generally sound but request major revisions to support the central conclusions. The authors must more rigorously justify the claim that *Sj*HAT1 is a viable and selective drug target by addressing conservation with host homologues, correcting the phylogenetic and domain analyses, and clarifying the proposed mechanism of action of DW-3-15. In addition, the transcriptomic and integrative components should be strengthened to present a more cohesive, hypothesis-driven study.

Reply：We thank the editors for their time spend making their constructive remarks and useful suggestions, which has significantly raised the quality of the manuscript and has enable us to improve the manuscript. If there were any other modifications we could make, we would like very much to modify them and we really appreciate your help. In response to the editors and reviewers’ feedback, we reconstructed the phylogenetic tree using an outgroup species and performed comprehensive structural domain analysis of *Sj*HAT1(Fig 1B). Our findings demonstrate that while *Sj*HAT1’s essential structural domains show remarkable evolutionary conservation, they display substantial spatial conformational divergence when compared with human HAT1. Molecular docking analysis reveals that *Sj*HAT1’s binding site for DW-3-15 is spatially distinct from that of *Hs*HAT1, accompanied by significant differences in surface electrostatic potential distribution (Fig 1C-1F). Collectively, these results suggest that despite preserving evolutionarily conserved core structural domains, *Sj*HAT1 exhibits unique three-dimensional architecture compared to its human ortholog, thereby reinforcing its potential as a promising therapeutic target for schistosomiasis intervention. We have revised Fig 6 with the addition of a heatmap visualizing differentially expressed genes and a corresponding gene list, aiming to enhance the clarity of our transcriptomic results.

**Fig 1. Multiple sequences alignment, phylogenetic analysis and molecular docking analyses of *Sj*HAT1.** (**A**) Multiple sequences alignment of *Sj*HAT1. Red box indicates the N-terminal domain of HAT1, blue box indicates the C-terminal domain of HAT1, and yellow triangles highlight key amino acids experimentally validated to be associated with HAT1 protein activity in other species (**B**) phylogenetic analysis of *Sj*HAT1. *Sj*HAT1 is shown in red. The right panel illustrates the structural organization and phylogenetic distribution of conserved motifs within HAT1 orthologs across different species. **(C)** Computational characterization of *Sj*HAT1/*Hs*HAT1and DW-3-15 interactions using molecular docking and ensemble analysis. Structural ensemble analysis was performed on the top 100 docking poses ranked by interface-predicted TM (ipTM) score through a computational modeling framework. Ligand conformations were chromatically coded according to binding energy gradients, while target proteins *Sj*HAT1/*Hs*HAT1 were displayed as monochromatic surface representations with transparency-adjusted secondary structures. **(D)** Three-dimensional structural model of *Sj*HAT1 with the highest ipTM score and its ligand-binding site. The DW-3-15 ligand represented as magenta sticks. Close-up view of the ligand-binding pocket, highlighting critical interactions with key amino acid residues (Trp-187, Tyr-215, Phe-217, Tyr-218, Tyr-220, Arg-227 and Glu-266). **(E)** Three-dimensional structural model of *Hs*HAT1 with the highest ipTM score and its ligand-binding site. The DW-3-15 ligand represented as pink sticks. Close-up view of the ligand-binding pocket, highlighting critical interactions with key amino acid residues (Gly-230, Gln-231, Ala-235, Glu-239, Lys-265, Leu-266, Asp-268, Phe-269, Ile-315). **(F)** Comparison of surface electrostatic potential distributions between *Sj*HAT1 and *Hs*HAT1 Proteins. Red areas denote negative electrostatic potential regions, blue areas correspond to positive electrostatic potential regions, and white areas represent neutral zones. The scale bar beneath indicates the corresponding electrostatic potential range (kT*e^-1^).

Fig 6. SjHAT1 regulates different pathways in female and male worms. (A) Volcano plot illustrating differentially expressed genes (DEGs) in female parasites. (B) Volcano plot illustrating DEGs in male worms. DEGs were defined as |log2 Fold Change| ≥0.58 and *p*-value< 0.05. (C) Heatmap of the differentially expressed genes. (D) KEGG pathway enrichment analysis of DEGs in female worms. (E) KEGG pathway enrichment analysis of DEGs in male worms. (F) Differentially expressed genes following *SjHAT1* knockdown. (G) RT-qPCR validation of *MRP4* (female) and *AADC* (male) expression. N = 3 bilogical replicates, Student’s *t*-test, ****p* < 0.001. Error bars represent standard deviation (SD).

**Journal Requirements:**

If the reviewer comments include a recommendation to cite specific previously published works, please review and evaluate these publications to determine whether they are relevant and should be cited. There is no requirement to cite these works unless the editor has indicated otherwise.

1) We do not publish any copyright or trademark symbols that usually accompany proprietary names, eg ©, ®, or TM (e.g. next to drug or reagent names). Therefore please remove all instances of trademark/copyright symbols throughout the text, including:

- TM on page: 24.

Reply：We thank the editor for pointing this error out, we have deleted the “TM” accordingly.

2) We have noticed that you have a list of Supporting Information legends in your manuscript. However, there are no corresponding files uploaded to the submission. Please upload them as separate files with the item type 'Supporting Information'.

Reply：We are sorry to make this mistake, and we have re-uploaded the ‘Supporting Information’ files.

3) Some material included in your submission may be copyrighted. According to PLOSu2019s copyright policy, authors who use figures or other material (e.g., graphics, clipart, maps) from another author or copyright holder must demonstrate or obtain permission to publish this material under the Creative Commons Attribution 4.0 International (CC BY 4.0) License used by PLOS journals. Please closely review the details of PLOSu2019s copyright requirements here: PLOS Licenses and Copyright. If you need to request permissions from a copyright holder, you may use PLOS's Copyright Content Permission form.

Please respond directly to this email and provide any known details concerning your material's license terms and permissions required for reuse, even if you have not yet obtained copyright permissions or are unsure of your material's copyright compatibility. Once you have responded and addressed all other outstanding technical requirements, you may resubmit your manuscript within Editorial Manager.

Potential Copyright Issues:

i) Please confirm (a) that you are the photographer of Figure 5C, 5E, S3B, S6D, 6C, and 6F, or (b) provide written permission from the photographer to publish the photo(s) under our CC BY 4.0 license.

Reply：We confirm that we are the photographer of Fig 2B, Fig 3F, Fig4, Fig 5C, 5E, 5G, Fig S3B, FigS4, FigS5, Fig S6C, S6D, and S5F.

4) Please amend your detailed Financial Disclosure statement. This is published with the article. It must therefore be completed in full sentences and contain the exact wording you wish to be published.

1) State the initials, alongside each funding source, of each author to receive each grant. For example: "This work was supported by the National Institutes of Health (####### to AM; ###### to CJ) and the National Science Foundation (###### to AM)."

2) State what role the funders took in the study. If the funders had no role in your study, please state: "The funders had no role in study design, data collection and analysis, decision to publish, or preparation of the manuscript."

3) If any authors received a salary from any of your funders, please state which authors and which funders.

If you did not receive any funding for this study, please simply state: The authors received no specific funding for this work.

Reply: We thank the editor for this kindly reminding, and we have amended our detailed ‘Financial Disclosure statement’ accordingly.

Reviewer #1:

1. *Schistosoma japonica* histone acetyltransferase 1, a novel anti-schistosomal drug target,” reports a well-designed project that comprehensively profiles the function of SjHAT1 and explores its potential as a drug target. The experiments are well designed, the statistics are appropriate, and the results are clear. While I have no major concerns with the experimental or methodological approach, I disagree with most of the interpretation and argument that SjHAT1 is a potential drug target. I also have questions about the composite understanding of SjHAT1 function in the context of previous work done by the authors exploring DW-3-15 as a compound that targets this HAT.

My primary critique is that based on the data reported here, I have the opposite conclusion as the authors regarding their interpretation of Sj-HAT1 being sufficiently diverged to be a good drug target. It's clear that the activity of the protein is important to proper development of the worm, and inhibition or knockdown of it seems to have strong deleterious effects on its fitness. However, this is not particularly surprising, given the importance of acetyltransferases on general developmental and physiological processes. While it's clear that the enzyme is important, the authors do not provide evidence that it can actually be targeted by a drug without off-target effects. In line 161, the authors state that there is only 34% identity, when in reality this is actually a fairly high value and indicates strong conservation with a human homologue. In line 164, based on the phylogenetic tree, the authors claim that SJ-hat-1 is in an evolutionarily distant clade, but the phylogenetic tree does not show that, and indeed cannot show that, given that it is unrooted with no outgroup. The authors repeat many of these claims in the discussion, such as lines 369 through 374, and suggest that molecules could be designed to target the unique domains of SJ, but their Figure 1B shows no such unique domains. Absent any other evidence from the authors, such as, for instance, experiments on DW-3-15's effects on mammalian acetyltransferases, or other protein modeling efforts, I interpret the author's own data in the exact opposite direction as they do - I'm convinced that SjHAT1 is not actually druggable.

Reply: We sincerely appreciate the reviewer’s insightful feedback regarding the limitations of sequence conservation analysis in our initial approach. Following your recommendation for rigorous phylogenetic reconstruction, we have reconstructed the phylogenetic tree by incorporating an outgroup species (Fig 1B). We anticipate that the revised phylogenetic tree will more clearly elucidate the evolutionary features of *Sj*HAT1.

To provide evidence that DW-3-15 specifically targets *Sj*HAT1 without off-target effects, we performed the *in vitro* experiments demonstrating that DW-3-15 has no effect on recombinant human HAT1. The corresponding results are shown in S1 Table. Our *in vivo* experiments also demonstrate that knocking down *SjHAT1* or inhibit its activity exerts prominent antischistosomal efficacy and protects the infected mice from severe progression of *Schistosoma japonicum* infection.

To be honest, a 34% identity is indeed relatively high for most species. However, in *Schistosoma japonicum*, there are 4,324 orthologs shared with *Homo sapiens*, representing 68% of its genome (6,322; PMID: 19606140). This high proportion makes it challenging to identify orthologs with very low homology to the human genome. Our previous analysis of the *Sj*HAT1 structural domain was relatively superficial and failed to comprehensively delineate its distinctions from homologous domains in humans. To address this, we performed an in-depth structural analysis of the *Sj*HAT1 using AlphaFold3 software. We anticipate these results will provide compelling evidence to support *Sj*HAT1 as a promising drug target. We predicted the tertiary structure of *Sj*HAT1 and performed a comparative analysis with its human homolog *Hs*HAT1. Structural superposition demonstrated that *Sj*HAT1 and *Hs*HAT1 share a conserved fold architecture, showing significant global structural similarity (TM-score = 0.92), but notable local divergence (RMSD = 2.40 Å). Molecular docking studies revealed that the *Sj*HAT1-DW-3-15 complex demonstrates heterogeneous binding modes, whereas the *Hs*HAT1-DW-3-15 complex exhibits limited conformational diversity (Fig 1C). Moreover, residue-level interaction analysis identified distinct binding patterns: *Sj*HAT1 engages the ligand primarily through Trp-187, Tyr-215, Phe-217, Tyr-218, Tyr-220, Arg-227, and Glu-266 (Fig 1D), whereas HsHAT1 interacts via Gly-230, Gln-231, Ala-235, Glu-239, Lys-265, Leu-266, Asp-268, Phe-269, and Ile-315 (Fig 1E). Electrostatic potential mapping revealed that *Sj*HAT1 possesses a significantly more electronegative binding cavity compared to *Hs*HAT1 (Fig 1F).  These findings indicate that although *Sj*HAT1 retains evolutionarily conserved core structural domains, it displays a distinctive three-dimensional conformation compared to its human ortholog. These distinct molecular characteristics, particularly the structural divergence from *Hs*HAT1, identifies this parasite enzyme as a viable candidate for anti-schistosomal drug discovery.

S1 Table Effect of DW-3-15 on the enzymatic activity of recombinant Human HAT1

| Group | ΔAFU | ΔT (min) | Acetyltransferase (HAT) activity (pmol/min)^*^ |
| --- | --- | --- | --- |
| Background | 0 | 30 | 0 |
| Positive control | 3006.3 | 30 | 10.3 |
| rHAT1^*^ (0.125 μg) | 10275.6 | 30 | 35.1 |
| rHAT1 (0.125 μg) +0.5 μM DW-3-15 | 10054.2 | 30 | 34.4 |
| rHAT1 (0.125 μg) +5 μM DW-3-15 | 10677.8 | 30 | 36.5 |
| rHAT1 (0.125 μg) +50 μM DW-3-15 | 10506.3 | 30 | 35.9 |

* rHAT1 activity (pmol/min) = ΔAFU/[time (min) * the slope of the standard curve].

ΔAFU= AFU(sample)-AFU(background). The regression equation of the Standard Curve is Y = 9.7537X + 131.71, R^2^=0.9967.

**Fig 1. Multiple sequences alignment, phylogenetic analysis and molecular docking analyses of *Sj*HAT1.** (**A**) Multiple sequences alignment of *Sj*HAT1. Red box indicates the N-terminal domain of HAT1, blue box indicates the C-terminal domain of HAT1, and yellow triangles highlight key amino acids experimentally validated to be associated with HAT1 protein activity in other species (**B**) phylogenetic analysis of *Sj*HAT1. *Sj*HAT1 is shown in red. The right panel illustrates the structural organization and phylogenetic distribution of conserved motifs within HAT1 orthologs across different species. **(C)** Computational characterization of *Sj*HAT1/*Hs*HAT1and DW-3-15 interactions using molecular docking and ensemble analysis. Structural ensemble analysis was performed on the top 100 docking poses ranked by interface-predicted TM (ipTM) score through a computational modeling framework. Ligand conformations were chromatically coded according to binding energy gradients, while target proteins *Sj*HAT1/*Hs*HAT1 were displayed as monochromatic surface representations with transparency-adjusted secondary structures. **(D)** Three-dimensional structural model of *Sj*HAT1 with the highest ipTM score and its ligand-binding site. The DW-3-15 ligand represented as magenta sticks. Close-up view of the ligand-binding pocket, highlighting critical interactions with key amino acid residues (Trp-187, Tyr-215, Phe-217, Tyr-218, Tyr-220, Arg-227 and Glu-266). **(E)** Three-dimensional structural model of *Hs*HAT1 with the highest ipTM score and its ligand-binding site. The DW-3-15 ligand represented as pink sticks. Close-up view of the ligand-binding pocket, highlighting critical interactions with key amino acid residues (Gly-230, Gln-231, Ala-235, Glu-239, Lys-265, Leu-266, Asp-268, Phe-269, Ile-315). **(F)** Comparison of surface electrostatic potential distributions between *Sj*HAT1 and *Hs*HAT1 Proteins. Red areas denote negative electrostatic potential regions, blue areas correspond to positive electrostatic potential regions, and white areas represent neutral zones. The scale bar beneath indicates the corresponding electrostatic potential range (kT*e^-1^).

2. My second primary comment is on the lack of clarity surrounding the hypothetical model of the mechanism of action of DW-3-15/SjHAT1 knockdown. Based on previous reports, SjHAT1 was proposed to be a target of DW-3-15 because it was identified as down-regulated by tandem mass spectrometry experiments. Here, authors show in an in vitro experiment with recombinant SjHAT1 that DW-3-15 acts on to inhibit its acetyltransferase activity (based on previous reviews). This experimental request is surprising, and the results are even more surprising, as the downregulation of a protein caused by a HAT inhibitor does not at all suggest that the protein is also itself the target of the molecule. Are the authors suggesting that DW-3-15 inhibits SjHAT1, which then leads to the downregulation of its own gene product? Are the authors aware of other examples in the literature of this proposed feedback loop where an epigenetic modulator acts on its own genetic locus? Based on the results from the multiple papers, it seems like this is the proposed mechanism of action of DW-3-15: SjHAT1 inhibition --> reduced acetylation --> chromatin condensation --> SjHAT1 downregulation --> deleterious effects.

Reply: We sincerely appreciate the reviewer’s insightful critique. It is true that downregulation of a protein caused by a HAT inhibitor does not necessarily imply that the protein itself is the inhibitor’s direct target. In order to address this fundamental concern, we performed *in vitro* and *in viv*o experiments to verify whether *Sj*HAT1 could be a promising target. First, we demonstrate that DW-3-15 downregulates the *Sj*HAT1expression in a dose-dependent manner both *in vitro* and *in vivo* (PMID: 39159234)*.* Second, RNAi-mediated *Sj*HAT1 knockdown induces phenotypic changes similar to DW-3-15. Downregulation of *Sj*HAT1 not only reduces worm survival but also suppresses female oviposition (Fig 3 and Fig 5). Meanwhile, silencing *Sj*HAT1 induces morphological changes similar to those observed after 72 h treatment with 100 μM DW-3-15 (Fig 4). These results indicate that *Sj*HAT1 is a potential drug target, although it may not be the sole target for DW-3-15.

Our experimental results demonstrate that DW-3-15 not only inhibits the enzymatic activity of *Sj*HAT1 but also downregulates its expression. These findings suggest that DW-3-15 influences both the activity and transcriptional levels of SjHAT1 through distinct pathways. However, further detailed studies are required to confirm this mechanism. This phenomenon is also evident in other multitarget compounds. For instance, curcumin exhibits dual effects: it directly inhibits COX-2 enzyme activity (PMID: 33540041) and indirectly suppresses COX-2 gene expression by modulating the ERK/NF-κB/COX-2 signaling pathway (PMID: 38004290).

3. Given the amount of work on this molecule and the target(s) over several published papers, I think that this manuscript would be a good place for the authors to summarize the current state of their working hypothesis for how DW-3-15 acts on SjHAT1 to have negative effects on schistosome fitness. The potential for other targets or non-specific events needs to be considered, especially since the molecule was shown to act on other stages while the experiments reported here focus on adults (and, particularly, females).

Reply: We thank the reviewer for this constructive suggestion. The reviewers’ perspectives are consistent with our research focus. Specifically, we are investigating the molecular mechanisms by which DW-3-15 induces inhibition or downregulation of *Sj*HAT1, leading to tegument damage in adult *S. japonicum*, and the mechanisms responsible for suppressing egg production in female schistosomes. However, due to the incomplete genome annotation of *S. japonicum* and the limited tools and methods available, progress has been relatively slow. Fortunately, breakthroughs have been made in the study of key molecular mechanisms regulating the reproductive development and egg-laying of *S. japonicum*. It has been reported that BATT secreted by male schistosomes is an essential pheromone that stimulates the maturation of the female reproductive system and egg production (https://doi.org/10.1016/j.cell.2022.03.017), with male aromatic L-amino acid decarboxylase (AADC) and non-ribosomal peptide synthetase (NRPS) identified as critical enzymes catalyzing BATT synthesis (PMID: 35294875, PMID: 38889152). Our research further revealed that downregulating *SjHAT1* significantly inhibits the expression of the male *AADC* gene and the female *multidrug resistance-associated protein 4* (*MRP4)* gene. MRPs are members of ABC transporters, and play a crucial role in signal transduction. Knockdown of SmMRP1 in *S. mansoni* disrupts egg production and mitigates liver pathology (https://doi.org/10.1371/journal.pntd.0001425). Taken together, we hypothesize that downregulation of *Sj*HAT1 inhibits the expression of key enzymes such as AADC in male worms, blocking the synthesis and excretion of BATT signals in males; by suppressing the ABC transporter system in female worms, it further hinders BATT signal transduction, thereby suppressing the development of the female reproductive system and oviposition.

Reviewer #1: Line 60: notbaly --> notably

Reply: We thank the reviewer for pointing out this mistake, we have corrected this error accordingly.

Line 253: bilogical --> biological

Reply: We thank the reviewer for pointing out this mistake, we have corrected this error accordingly.

Line 312: comparation --> comparison

Reply: We thank the reviewer for pointing out this mistake, we have corrected this error accordingly.

Line 313: replicates --> replicate

Reply: We thank the reviewer for pointing out this mistake, we have corrected this error accordingly.

Line 375: genders --> sexes

Reply: We thank the reviewer for revising ‘genders’ to ‘sexes’, we have corrected accordingly.

Line 414: italics on in vitro

Reply: Already done.

PRJNA1313613 is not available on NIH BioProject

Reply: All data have been deposited in the NIH BioProject under a three-year embargo, with public release scheduled for 2028. Reviewers can access the data via https://dataview.ncbi.nlm.nih.gov/object/PRJNA1313613?reviewer=osmnag1rm66sc4llt995mg05tp in read-only format.

Figure 5C - Given that the images are available, is it possible to get quantitative measurements? The qualitative images are not entirely convincing.

Reply: We thank the reviewer for this constructive suggestion. After interference with *SjHAT1*-dsRNA, the female worms failed to mature and remained significantly smaller in size. Hence, collecting these worms via hepatic portal vein perfusion became a challenging process. At the time, our primary focus was on accurately quantifying the worm burden, which led to inadequate preservation of some specimens. Consequently, we were unable to obtain length measurements for all female worms. To mitigate this limitation, we subsequently documented the collected specimens photographically, as presented in the current figures. We acknowledge this methodological constraint with regret.

Reviewer #2:

1. The phylogenetic analysis is misinterpreted or misconstrued. The tree shows the evolution that is expected of a vertically inherited schistomsome gene. I think it is fine to report it, the issue is just the result are conveyed as being unusual, when in fact they are quite ordinary.

Reply: We thank the reviewer for this constructive suggestion. We may have initially overinterpreted the phylogenetic tree results; therefore, we conducted additional molecular docking analyses to confirm the structural specificity of the *Sj*HAT1 binding pocket.

2. Figure 6 is unnecessary. The results are summarized in the text and nothing new is added. At most they could be summarized in a table. But I think it could be improved by replacing it with a heat map of the differentially expressed genes that shows the regulation and has labels that highlight genes in pathways discussed in manuscript.

Reply: We thank the reviewer for this constructive suggestion. We have revised Figure 6 by incorporating a heatmap, aiming to provide a clearer demonstration of our results.

Fig 6. SjHAT1 regulates different pathways in female and male worms. (A) Volcano plot illustrating differentially expressed genes (DEGs) in female parasites. (B) Volcano plot illustrating DEGs in male worms. DEGs were defined as |log2 Fold Change| ≥0.58 and p-value< 0.05. (C) Heatmap of the differentially expressed genes. (D) KEGG pathway enrichment analysis of DEGs in female worms. (E) KEGG pathway enrichment analysis of DEGs in male worms. (F) Differentially expressed genes following *SjHAT1* knockdown. (G) qRT-PCR validation of *MRP4* (female) and *AADC* (male) expression. N = 3 bilogical replicates, Student’s *t*-test, ****p* < 0.001. Error bars represent standard deviation (SD).

Figure resubmission:

While revising your submission, we strongly recommend that you use PLOS’s NAAS tool (https://ngplosjournals.pagemajik.ai/artanalysis) to test your figure files. NAAS can convert your figure files to the TIFF file type and meet basic requirements (such as print size, resolution), or provide you with a report on issues that do not meet our requirements and that NAAS cannot fix.

After uploading your figures to PLOS’s NAAS tool - https://ngplosjournals.pagemajik.ai/artanalysis, NAAS will process the files provided and display the results in the "Uploaded Files" section of the page as the processing is complete. If the uploaded figures meet our requirements (or NAAS is able to fix the files to meet our requirements), the figure will be marked as "fixed" above. If NAAS is unable to fix the files, a red "failed" label will appear above. When NAAS has confirmed that the figure files meet our requirements, please download the file via the download option, and include these NAAS processed figure files when submitting your revised manuscript.

Reply: We thank the editor for this constructive suggestion. We have carefully checked all the Figures using NAAS, and they all comply with the requirements.

Reproducibility:

To enhance the reproducibility of your results, we recommend that authors of applicable studies deposit laboratory protocols in protocols.io, where a protocol can be assigned its own identifier (DOI) such that it can be cited independently in the future. Additionally, PLOS ONE offers an option to publish peer-reviewed clinical study protocols. Read more information on sharing protocols at <https://plos.org/protocols?utm_medium=editorial-email&utm_source=authorletters&utm_campaign=protocols>

Reply: We thank the editor for this constructive suggestion. If necessary, we will deposit our laboratory protocols in protocols.io.
