# Supplementary material for: Schistosoma japonicum histone acetyltransferase 1 (SjHAT1): A novel anti-schistosomal drug target
Source: PLoS Pathog. 2026 Jun 24;22(6):e1014334. doi: 10.1371/journal.ppat.1014334 (PMC13293438; doi:10.1371/journal.ppat.1014334)
Supplement: Peer review history

# Author comment: Author response to Decision Letter 0

**DOI:** 10.1371/journal.ppat.1014334.r001

22 Dec 2025

Attachment Submitted filename: Response to reviewers.docx

# Peer review: Decision Letter 0

**DOI:** 10.1371/journal.ppat.1014334.r002

**Authors:** Dominique Soldati-Favre, Mostafa Zamanian

25 Feb 2026

PPATHOGENS-D-25-03238

Schistosoma japonicum Histone Acetyltransferase 1 (SjHAT1): A Novel Anti-schistosomal Drug Target

PLOS Pathogens

Dear Dr. Xia,

Thank you for submitting your manuscript to PLOS Pathogens. After careful consideration, we feel that it has merit but does not fully meet PLOS Pathogens's publication criteria as it currently stands. Therefore, we invite you to submit a revised version of the manuscript that addresses the points raised during the review process.

Please submit your revised manuscript by Apr 26 2026 11:59PM. If you will need more time than this to complete your revisions, please reply to this message or contact the journal office at plospathogens@plos.org. When you're ready to submit your revision, log on to https://www.editorialmanager.com/ppathogens/ and select the 'Submissions Needing Revision' folder to locate your manuscript file.

Please include the following items when submitting your revised manuscript:

* A letter that responds to each point raised by the editor and reviewer(s). You should upload this letter as a separate file labeled 'Response to Reviewers'. This file does not need to include responses to any formatting updates and technical items listed in the 'Journal Requirements' section below.

* A marked-up copy of your manuscript that highlights changes made to the original version. You should upload this as a separate file labeled 'Revised Manuscript with Track Changes'.

* An unmarked version of your revised paper without tracked changes. You should upload this as a separate file labeled 'Manuscript'.

If you would like to make changes to your financial disclosure, competing interests statement, or data availability statement, please make these updates within the submission form at the time of resubmission. Guidelines for resubmitting your figure files are available below the reviewer comments at the end of this letter.

We look forward to receiving your revised manuscript.

Kind regards,

Mostafa Zamanian

Academic Editor

PLOS Pathogens

Dominique Soldati-Favre

Section Editor

PLOS Pathogens

Sumita Bhaduri-McIntosh

Editor-in-Chief

PLOS Pathogens

orcid.org/0000-0003-2946-9497

Michael Malim

Editor-in-Chief

PLOS Pathogens

orcid.org/0000-0002-7699-2064

**Additional Editor Comments:**

The reviewers find the experimental work generally sound but request major revisions to support the central conclusions. The authors must more rigorously justify the claim that SjHAT1 is a viable and selective drug target by addressing conservation with host homologues, correcting the phylogenetic and domain analyses, and clarifying the proposed mechanism of action of DW-3-15. In addition, the transcriptomic and integrative components should be strengthened to present a more cohesive, hypothesis-driven study.

**Journal Requirements:**

If the reviewer comments include a recommendation to cite specific previously published works, please review and evaluate these publications to determine whether they are relevant and should be cited. There is no requirement to cite these works unless the editor has indicated otherwise.

1) We do not publish any copyright or trademark symbols that usually accompany proprietary names, eg ©,  ®, or TM  (e.g. next to drug or reagent names). Therefore please remove all instances of trademark/copyright symbols throughout the text, including:

- TM on page: 24.

2) We have noticed that you have a list of Supporting Information legends in your manuscript. However, there are no corresponding files uploaded to the submission. Please upload them as separate files with the item type 'Supporting Information'.

3) Some material included in your submission may be copyrighted. According to PLOSu2019s copyright policy, authors who use figures or other material (e.g., graphics, clipart, maps) from another author or copyright holder must demonstrate or obtain permission to publish this material under the Creative Commons Attribution 4.0 International (CC BY 4.0) License used by PLOS journals. Please closely review the details of PLOSu2019s copyright requirements here: PLOS Licenses and Copyright. If you need to request permissions from a copyright holder, you may use PLOS's Copyright Content Permission form.

Please respond directly to this email and provide any known details concerning your material's license terms and permissions required for reuse, even if you have not yet obtained copyright permissions or are unsure of your material's copyright compatibility. Once you have responded and addressed all other outstanding technical requirements, you may resubmit your manuscript within Editorial Manager.

Potential Copyright Issues:

i) Please confirm (a) that you are the photographer of S2B, S5D, 5C, and 5E, or (b) provide written permission from the photographer to publish the photo(s) under our CC BY 4.0 license.

4) Please amend your detailed Financial Disclosure statement. This is published with the article. It must therefore be completed in full sentences and contain the exact wording you wish to be published.

1) State the initials, alongside each funding source, of each author to receive each grant. For example: "This work was supported by the National Institutes of Health (####### to AM; ###### to CJ) and the National Science Foundation (###### to AM)."

2) State what role the funders took in the study. If the funders had no role in your study, please state: "The funders had no role in study design, data collection and analysis, decision to publish, or preparation of the manuscript."

3) If any authors received a salary from any of your funders, please state which authors and which funders..

If you did not receive any funding for this study, please simply state: u201cThe authors received no specific funding for this work.u201d

**Reviewers' Comments:**

Reviewer's Responses to Questions

**Part I - Summary**

Please use this section to discuss strengths/weaknesses of study, novelty/significance, general execution and scholarship.

Reviewer #1: "Schistosoma japonica histone acetyltransferase 1, a novel anti-schistosomal drug target," reports a well-designed project that comprehensively profiles the function of SjHAT1 and explores its potential as a drug target. The experiments are well designed, the statistics are appropriate, and the results are clear. While I have no major concerns with the experimental or methodological approach, I disagree with most of the interpretation and argument that SjHAT1 is a potential drug target. I also have questions about the composite understanding of SjHAT1 function in the context of previous work done by the authors exploring DW-3-15 as a compound that targets this HAT.

My primary critique is that based on the data reported here, I have the opposite conclusion as the authors regarding their interpretation of SJ-HAT1 being sufficiently diverged to be a good drug target. It's clear that the activity of the protein is important to proper development of the worm, and inhibition or knockdown of it seems to have strong deleterious effects on its fitness. However, this is not particularly surprising, given the importance of acetyl transferases on general developmental and physiological processes. While it's clear that the enzyme is important, the authors do not provide evidence that it can actually be targeted by a drug without off-target effects. In line 161, the authors state that there is only 34% identity, when in reality this is actually a fairly high value and indicates strong conservation with a human homologue. In line 164, based on the phylogenetic tree, the authors claim that SJ-hat-1 is in an evolutionarily distant clade, but the phylogenetic tree does not show that, and indeed cannot show that, given that it is unrooted with no outgroup. The authors repeat many of these claims in the discussion, such as lines 369 through 374, and suggest that molecules could be designed to target the unique domains of SJ, but their Figure 1B shows no such unique domains. Absent any other evidence from the authors, such as, for instance, experiments on DW-3-15's effects on mammalian acetyltransferases, or other protein modeling efforts, I interpret the author's own data in the exact opposite direction as they do - I'm convinced that SjHAT1 is not actually druggable.

My second primary comment is on the lack of clarity surrounding the hypothetical model of the mechanism of action of DW-3-15/SjHAT1 knockdown. Based on previous reports, SjHAT1 was proposed to be a target of DW-3-15 because it was identified as down-regulated by tandem mass spectrometry experiments. Here, authors show in an in vitro experiment with recombinant SjHAT1 that DW-3-15 acts on to inhibit its acetyltransferase activity (based on previous reviews). This experimental request is surprising, and the results are even more surprising, as the downregulation of a protein caused by a HAT inhibitor does not at all suggest that the protein is also itsef the target of the molecule. Are the authors suggesting that DW-3-15 inhibits SjHAT1, which then leads to the downregulation of its own gene product? Are the authors aware of other examples in the literature of this proposed feedback loop where an epigenetic modulator acts on its own genetic locus? Based on the results from the multiple papers, it seems like this is the proposed mechanism of action of DW-3-15: SjHAT1 inhibition --> reduced acetylation --> chromatin condensation --> SjHAT1 downregulation --> deleterious effects. Given the amount of work on this molecule and the target(s) over several published papers, I think that this manuscript would be a good place for the authors to summarize the current state of their working hypothesis for how DW-3-15 acts on SjHAT1 to have negative effects on schistosome fitness. The potential for other targets or non-specific events needs to be considered, especially since the molecule was shown to act on other stages while the experiments reported here focus on adults (and, particularly, females).

Reviewer #2: The manuscript focuses on Schistosoma japonicum Histone Acetyltransferase (SjHAT1): A Novel Anti-schistosomal Drug Target. The authors have already demonstrated in prior work that a praziquantel derivative acts though SjHAT1 inhibition. In the current work they cloned SjHAT1, showed that it is active in acetylating histones and that it is inhibited by the drug they have described previously. They conducted a phylogenetic analysis, showed stage and sex-specific patterns with qRT-PCR, and examine localization using microscopy including FISH and EM. Lastly, they undertook an RNA sequencing analysis comparing knockdown to control. Overall their data suggests that downstream effects are on pheromone signaling and reproduction.

**********

**Part II – Major Issues: Key Experiments Required for Acceptance**

Please use this section to detail the key new experiments or modifications of existing experiments that should be absolutely required to validate study conclusions.

Generally, there should be no more than 3 such required experiments or major modifications for a "Major Revision" recommendation. If more than 3 experiments are necessary to validate the study conclusions, then you are encouraged to recommend "Reject".

Reviewer #1: NA

Reviewer #2: 1. The phylogenetic analysis is misinterpreted or misconstrued. The tree shows the evolution that is expected of a vertically inherited schistomsome gene. I think it is fine to report it, the issue is just the result are conveyed as being unusual, when in fact they are quite ordinary.

2. Figure 6 is unnecessary. The results are summarized in the text and nothing new is added. At most they could be summarized in a table. But I think it could be improved by replacing it with a heat map of the differentially expressed genes that shows the regulation and has labels that highlight genes in pathways discussed in manuscript.

**********

**Part III – Minor Issues: Editorial and Data Presentation Modifications**

Please use this section for editorial suggestions as well as relatively minor modifications of existing data that would enhance clarity.

Reviewer #1: Line 60: notbaly --> notably

Line 253: bilogical --> biological

Line 312: comparation --> comparison

Line 313: replicates --> replicate

Line 375: genders --> sexes

Line 414: italics on in vitro

PRJNA1313613 is not available on NIH BioProject

Figure 5C - Given that the images are available, is it possible to get quantitative measurements? The qualitative images are not entirely convincing.

Reviewer #2: (No Response)

**********

PLOS authors have the option to publish the peer review history of their article (what does this mean?). If published, this will include your full peer review and any attached files.

If you choose “no”, your identity will remain anonymous but your review may still be made public.

**Do you want your identity to be public for this peer review?**   For information about this choice, including consent withdrawal, please see our Privacy Policy.

Reviewer #1: No

Reviewer #2: No

**Figure resubmission:**

-->While revising your submission, we strongly recommend that you use PLOS’s NAAS tool (https://ngplosjournals.pagemajik.ai/artanalysis) to test your figure files. NAAS can convert your figure files to the TIFF file type and meet basic requirements (such as print size, resolution), or provide you with a report on issues that do not meet our requirements and that NAAS cannot fix.

After uploading your figures to PLOS’s NAAS tool - https://ngplosjournals.pagemajik.ai/artanalysis, NAAS will process the files provided and display the results in the "Uploaded Files" section of the page as the processing is complete. If the uploaded figures meet our requirements (or NAAS is able to fix the files to meet our requirements), the figure will be marked as "fixed" above. If NAAS is unable to fix the files, a red "failed" label will appear above. When NAAS has confirmed that the figure files meet our requirements, please download the file via the download option, and include these NAAS processed figure files when submitting your revised manuscript.

**Reproducibility:**

To enhance the reproducibility of your results, we recommend that authors of applicable studies deposit laboratory protocols in protocols.io, where a protocol can be assigned its own identifier (DOI) such that it can be cited independently in the future. Additionally, PLOS ONE offers an option to publish peer-reviewed clinical study protocols. Read more information on sharing protocols at https://plos.org/protocols?utm_medium=editorial-email&utm_source=authorletters&utm_campaign=protocols

# Author comment: Author response to Decision Letter 1

**DOI:** 10.1371/journal.ppat.1014334.r003

30 Apr 2026

Attachment Submitted filename: Response to Reviewers-new.docx

# Peer review: Decision Letter 1

**DOI:** 10.1371/journal.ppat.1014334.r004

**Authors:** Dominique Soldati-Favre, Mostafa Zamanian, Dominique Soldati-Favre, Mostafa Zamanian

2 Jun 2026

Dear Prof. Xia,

We are pleased to inform you that your manuscript 'Schistosoma japonicum Histone Acetyltransferase 1 (SjHAT1): A Novel Anti-schistosomal Drug Target' has been provisionally accepted for publication in PLOS Pathogens.

Before your manuscript can be formally accepted you will need to complete some formatting changes, which you will receive in a follow up email. A member of our team will be in touch with a set of requests.

Please note that your manuscript will not be scheduled for publication until you have made the required changes, so a swift response is appreciated.

IMPORTANT: The editorial review process is now complete. PLOS will only permit corrections to spelling, formatting or significant scientific errors from this point onwards. Requests for major changes, or any which affect the scientific understanding of your work, will cause delays to the publication date of your manuscript.

Should you, your institution's press office or the journal office choose to press release your paper, you will automatically be opted out of early publication. We ask that you notify us now if you or your institution is planning to press release the article. All press must be co-ordinated with PLOS.

Thank you again for supporting Open Access publishing; we are looking forward to publishing your work in PLOS Pathogens.

Best regards,

Mostafa Zamanian

Academic Editor

PLOS Pathogens

Dominique Soldati-Favre

Section Editor

PLOS Pathogens

Sumita Bhaduri-McIntosh

Editor-in-Chief

PLOS Pathogens

orcid.org/0000-0003-2946-9497

Michael Malim

Editor-in-Chief

PLOS Pathogens

orcid.org/0000-0002-7699-2064

***********************************************************

Reviewer Comments (if any, and for reference):

Reviewer's Responses to Questions

**Part I - Summary**

Please use this section to discuss strengths/weaknesses of study, novelty/significance, general execution and scholarship.

Reviewer #1: I thank the authors for their revisions and congratulate them on a compelling project.

**********

**Part II – Major Issues: Key Experiments Required for Acceptance**

Please use this section to detail the key new experiments or modifications of existing experiments that should be absolutely required to validate study conclusions.

Generally, there should be no more than 3 such required experiments or major modifications for a "Major Revision" recommendation. If more than 3 experiments are necessary to validate the study conclusions, then you are encouraged to recommend "Reject".

Reviewer #1: (No Response)

**********

**Part III – Minor Issues: Editorial and Data Presentation Modifications**

Please use this section for editorial suggestions as well as relatively minor modifications of existing data that would enhance clarity.

Reviewer #1: (No Response)

**********

PLOS authors have the option to publish the peer review history of their article (what does this mean?). If published, this will include your full peer review and any attached files.

If you choose “no”, your identity will remain anonymous but your review may still be made public.

**Do you want your identity to be public for this peer review?**   For information about this choice, including consent withdrawal, please see our Privacy Policy.

Reviewer #1: No

# Editor report: Acceptance letter

**DOI:** 10.1371/journal.ppat.1014334.r005

**Authors:** Dominique Soldati-Favre, Mostafa Zamanian, Dominique Soldati-Favre, Mostafa Zamanian

Dear Prof. Xia,

We are delighted to inform you that your manuscript, "Schistosoma japonicum Histone Acetyltransferase 1 (SjHAT1): A Novel Anti-schistosomal Drug Target," has been formally accepted for publication in PLOS Pathogens.

We have now passed your article onto the PLOS Production Department who will complete the rest of the pre-publication process. All authors will receive a confirmation email upon publication.

The corresponding author will soon be receiving a typeset proof for review, to ensure errors have not been introduced during production. Please review the PDF proof of your manuscript carefully, as this is the last chance to correct any scientific or type-setting errors. Please note that major changes, or those which affect the scientific understanding of the work, will likely cause delays to the publication date of your manuscript. Note: Proofs for Front Matter articles (Pearls, Reviews, Opinions, etc...) are generated on a different schedule and may not be made available as quickly.

Soon after your final files are uploaded, the early version of your manuscript, if you opted to have an early version of your article, will be published online. The date of the early version will be your article's publication date. The final article will be published to the same URL, and all versions of the paper will be accessible to readers.

For Research Articles, you will receive an invoice from PLOS for your publication fee after your manuscript has reached the completed accept phase. If you receive an email requesting payment before acceptance or for any other service, this may be a phishing scheme. Learn how to identify phishing emails and protect your accounts at https://explore.plos.org/phishing.

Thank you again for supporting open-access publishing; we are looking forward to publishing your work in PLOS Pathogens.

Best regards,

Sumita Bhaduri-McIntosh

Editor-in-Chief

PLOS Pathogens

orcid.org/0000-0003-2946-9497

Michael Malim

Editor-in-Chief

PLOS Pathogens

orcid.org/0000-0002-7699-2064
